# Layer‐by‐layer nanoparticles for novel delivery of cisplatin and PARP inhibitors for platinum‐based drug resistance therapy in ovarian cancer

**DOI:** 10.1002/btm2.10131

**Published:** 2019-06-14

**Authors:** Lawrence B. Mensah, Stephen W. Morton, Jiahe Li, Haihua Xiao, Mohiuddin A. Quadir, Kevin M. Elias, Emily Penn, Aysen K. Richson, Paiman Peter Ghoroghchian, Joyce Liu, Paula T. Hammond

**Affiliations:** ^1^ The Koch Institute for Integrative Cancer Research Massachusetts Institute of Technology (MIT) Cambridge MA, 02142; ^2^ Department of Chemical Engineering Massachusetts Institute of Technology (MIT) Cambridge MA, 02139; ^3^ Institute of Chemistry, Changchun Institute of Applied Chemistry Chinese Academy of Sciences, Jilin Changchun P.R. China; ^4^ Department of Coatings and Polymeric Materials North Dakota State University Fargo ND, 58108; ^5^ Division of Gynecologic Oncology, Department of Obstetrics and Gynecology, and Reproductive Biology Brigham and Women's Hospital Boston MA, 02115; ^6^ Dana‐Farber Cancer Institute Boston MA, 02115

**Keywords:** BMN 673, cisplatin, layer‐by‐layer, nanomedicine, nanoparticles, olaparib, ovarian cancer, PARP inhibitors

## Abstract

Advanced staged high‐grade serous ovarian cancer (HGSOC) is the leading cause of gynecological cancer death in the developed world, with 5‐year survival rates of only 25–30% due to late‐stage diagnosis and the shortcomings of platinum‐based therapies. A Phase I clinical trial of a combination of free cisplatin and poly(ADP‐ribose) polymerase inhibitors (PARPis) showed therapeutic benefit for HGSOC. In this study, we address the challenge of resistance to platinum‐based therapy by developing a targeted delivery approach. Novel electrostatic layer‐by‐layer (LbL) liposomal nanoparticles (NPs) with a terminal hyaluronic acid layer that facilitates CD44 receptor targeting are designed for selective targeting of HGSOC cells; the liposomes can be formulated to contain both cisplatin and the PARPi drug within the liposomal core and bilayer. The therapeutic effectiveness of LbL NP‐encapsulated cisplatin and PARPi alone and in combination was compared with the corresponding free drugs in luciferase and CD44‐expressing OVCAR8 orthotopic xenografts in female nude mice. The NPs exhibited prolonged blood circulation half‐life, mechanistic staged drug release and targeted codelivery of the therapeutic agents to HGSOC cells. Moreover, compared to the free drugs, the NPs resulted in significantly reduced tumor metastasis, extended survival, and moderated systemic toxicity.

## 1 INTRODUCTION

Epithelial ovarian cancer (EOC) is the second most common gynecological cancer and the leading cause of death from gynecologic malignancies in the developed world.^1^, ^2^ High‐grade serous ovarian cancer (HGSOC) accounts for more than 70–80% of ovarian cancer‐associated mortalities.^2^, ^3^ This high mortality rate is attributable to an aggressive phenotype, diagnosis at advanced stages, and the development of resistance against mainstay platinum‐based therapies.^4^ Cisplatin and other platinum‐based chemotherapies efficiently bind and induce DNA double‐strand breaks (DSBs) and apoptosis in cancer cells^5^ and are the current cytotoxic drugs of choice for ovarian cancer and other carcinomas.^6^, ^7^ However, the rapid development of resistance often limits the effectiveness of platinum‐based drugs alone against solid tumors such as HGSOC.^8^, ^9^

Among solid tumors, 15–46% of HGSOC, 40–66% of triple‐negative breast cancer, and 2–9% of non‐small cell lung carcinoma are estimated to carry mutations in the *p53*, *BRCA1*, *BRCA2*, and *PTEN* genes, which are required for DNA damage repair via homologous recombination (HR).^5^ Consequently, using DNA‐damaging agents in combination with inhibitors of DNA damage repair proteins is a very attractive strategy. In the past 5 years, new classes of inhibitors have emerged against poly(ADP‐ribose) polymerases (PARPs), a family of nuclear DNA damage repair enzymes with a role in the maintenance of genomic stability.^10^ PARPs perform this function by initiating base excision repair and nucleotide excision repair of DNA single‐strand breaks (SSBs).^11^ The inhibition of SSB repair by PARP inhibitors (PARPis) induces and confers sensitivity and synthetic lethality to cells with defective HR‐directed DSB repair.^11^, ^12^ PARPis exhibit synergistic activity when combined with a DNA‐damaging agent by interfering with DNA repair and potentiating the activity of the chemotherapeutic agent. The potentiation effect is achieved via inhibition of the catalytic activity of PARP by PARPis, or by trapping PARP at SSB sites, thereby stalling the replication fork and DNA transcription^10^, ^11^ and eventually leading to apoptosis.

Different classes of PARPis of varying toxicity and efficacy have been developed.^13^, ^14^ Of the five most clinically relevant PARPis, three of them: AZD2281 (olaparib, Lynparza; AstraZeneca, UK),^15^ niraparib (Zejula, MK4827 Tesaro, Waltham, MA),^16^ and rucaparib (Rubraca; Clovis Oncology, Boulder, CO)^17^ are FDA approved for the treatment of recurrent EOC. BMN 673 and veliparib are under investigation in different phases of clinical trials.^10^, ^11^

The ability of DNA damaging agents to enhance apoptosis and reduce drug resistance in HR‐deficient cells in tumors has led to a number of preclinical investigations. Rottenberg et al.^18^ and Hay et al.^19^ showed that the free‐drug combination of AZD2281 with cisplatin or carboplatin significantly reduced resistance to platinum‐based agents in *BRCA1* mutated ovarian and breast cancer tumor‐bearing mice and prolonged overall survival compared with either monotherapy. Others studies have shown high tolerance for AZD2281 alone but not in combination with other chemotherapies.^18^ Several Phase I–III clinical trials have been conducted to evaluate AZD2281 in combination with cisplatin and other chemotherapies in advanced breast and ovarian cancers in patients with *BRCA* mutation.^20^, ^21^ Overall, the data indicated that the high‐dose combination of cisplatin with AZD2281 was not tolerable in most patients. However, a moderate dose of cisplatin (60 mg/m^2^) and AZD2281 (50 mg/twice daily) was better tolerated in most patients. In addition, the AZD2281 and cisplatin combination prolonged progression‐free survival in patients compared to monotherapy, with tolerable side effects.^20^, ^22^

BMN 673 (talazoparib) remains one of the most promising PARP1/2 inhibitors, and we have also tested BMN 673 alongside AZD2281 as monotherapies or in combination with cisplatin.^23^ Preclinical testing has shown that BMN 673 exhibits superior PARP inhibition and antitumor activity in vitro^24^, ^25^, ^26^, ^27^ and in vivo.^28^ A number of completed Phase I and II clinical trials of BMN 673 have evaluated its tolerability, efficacy, pharmacokinetics, and safety in both ovarian and metastatic breast cancer^24^, ^29^ and Phase III clinical trials are currently underway.^11^ In Phase I and II clinical trials, the combination of BMN 673 with carboplatin showed synergy and significant therapeutic effects. However, hematologic toxicity was pronounced, particularly in g*BRCA* patients.^29^ The clinical benefit of BMN 673 was 56–86% in both breast and ovarian cancer patients, with higher efficacy for the combination with carboplatin.^29^

Although combinations of PARPis with cisplatin are efficacious, these preclinical and clinical trials of AZD2281 and BMN 673 alone or in combination with chemotherapies have revealed a number of hurdles that remain to be overcome to harness their full antitumor potential in the clinical setting. First, PARPis are highly hydrophobic, with limited bioavailability and a relatively rapid plasma clearance rate. Rothenberg et al.^18^ described rapid plasma clearance of AZD2281 when delivered in free form in tumor‐bearing mouse models. Second, cisplatin, which remains a key platinum agent for ovarian cancer therapy, is subject to the development of resistance in tumors and therefore is typically administered at a high dose in the clinic, leading to its well‐known systemic toxicity.^25^, ^26^ Third, the therapeutic combination of cisplatin and AZD2281 is poorly tolerated in patients due to the overlapping toxicities of the two drugs^27^; hence, only reduced doses have been evaluated in clinical trials. Fourth, the infusion and oral routes of administration of cisplatin and PARPis, respectively, can reduce medication compliance, leading to a less effective therapeutic response in patients. Moreover, to obtain the highest therapeutic index, both cisplatin and PARPi should be codelivered at their highest doses and at an appropriate therapeutic ratio to tumors, which is difficult to achieve via conventional free drug delivery approaches. Finally, the two drugs have different biodistribution profiles when administered via different routes by traditional approach. These factors affect the time it takes each drug to reach the tumor and the drug concentration delivered and can significantly affect treatment outcomes. Codelivery of these drug combinations via a nanocarrier approach could significantly reduce or eliminate these hurdles.^30^, ^31^

Advances in nanotechnology and nanomedicine have provided new opportunities for synergistic combinations of therapeutic agents via single multicompartment nanoparticles (NPs).^32^, ^33^, ^34^, ^35^ The three main goals of this study are to (a) address the unmet clinical need for an effective and safe platform for the delivery of combination therapies to ovarian cancer; (b) design safe, full‐dose delivery of cisplatin, and PARPis to overcome cisplatin drug resistance in ovarian cancer therapy; (c) evaluate potential systemic toxicity associated with this treatment platform. We describe a novel approach to provide safe therapeutic delivery of cisplatin and PARPis to tumors using the layer‐by‐layer (LbL) polymeric liposomal NPs approach. These NPs achieve synergistic drug delivery while inherently addressing many of the challenges associated with the conventional delivery of cocktails of free drugs, such as lack of targeted mechanistic delivery, reduced drug blood circulation, and the use of dual routes.^32^, ^36^, ^37^ The HA‐terminated outer layer of the LbL NPs enables CD44 receptor targeting on HGSOC tumors, while the pH‐responsive poly(_L_‐lysine) (PLL) layer facilitates tunable intracellular release of the therapeutic cargo in tumor cells.^36^, ^38^

We report the novel packaging of cisplatin with AZD2281 or BMN 673 in LbL NPs for orthotopic HGSOC therapy. We also perform a head‐to‐head comparison of the therapeutic efficacy of both free and nano‐encapsulated delivery of AZD2281 and BMN 673 in vivo as a single maintenance agent in orthotopic HGSOC tumor‐bearing mice. In summary, we observed an overall increase in survival and improved treatment outcomes in mice treated with the LbL‐encapsulated drug combination compared with free drug combination therapy.^39^, ^40^

## 2 RESULTS

### 2.1 LbL polymeric liposomal NPs exhibit controlled drug release and inhibit HR

**Figure 1:**
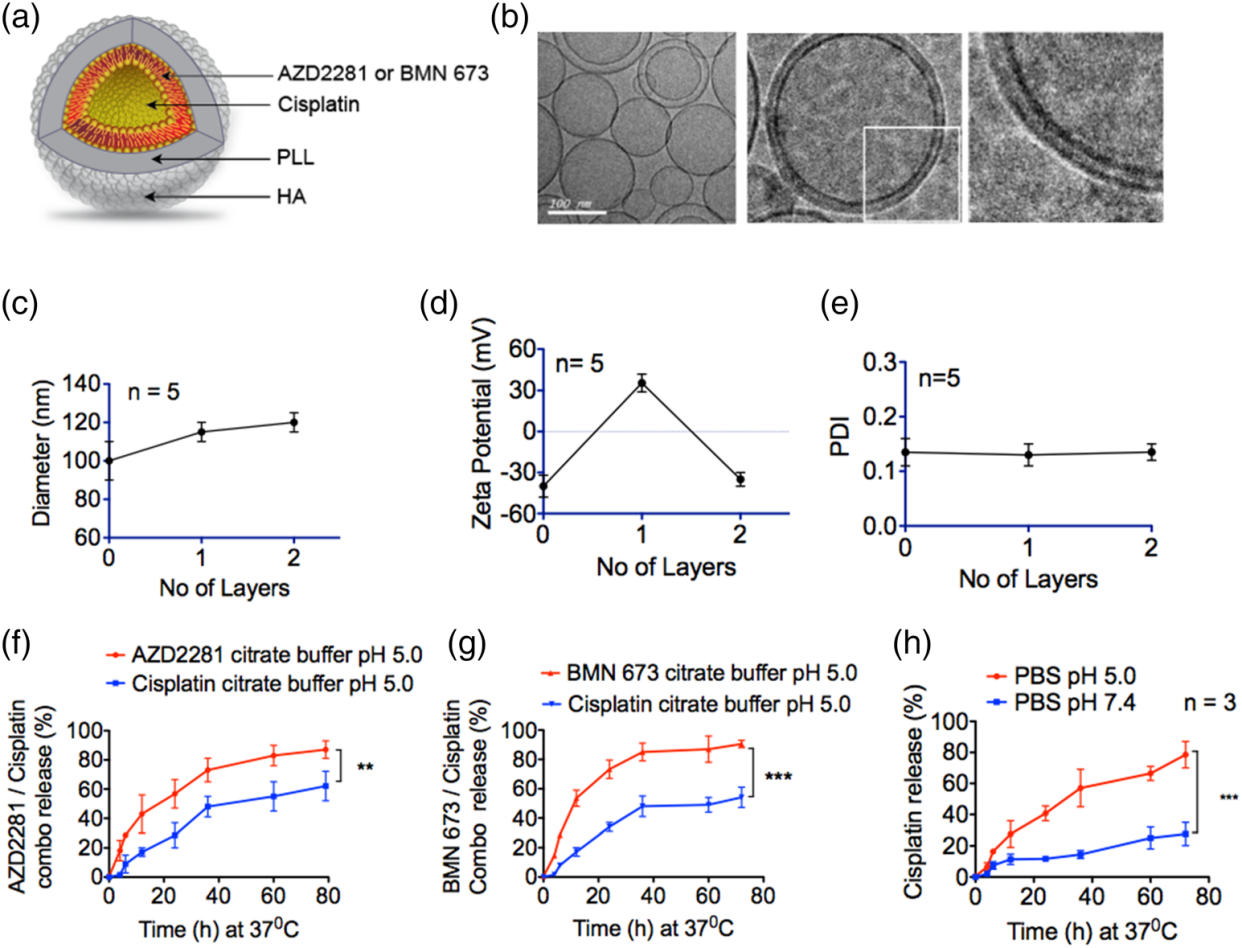
Physiochemical characterization of the polymeric liposomal nanoparticles. (a) Illustration of the polymeric liposomal nanoparticle formulated by lipid self‐assembly and layered with a polycation, poly(_L_‐lysine) (PLL), and a terminal hyaluronic acid (HA) layer for CD44 targeting. The PARPi AZD2281 or BMN 673 was loaded into the liposomes lipid bilayer, and cisplatin was loaded into the core. (b) Electron micrographs of the nanoparticles. Left panel, liposomes alone. Middle panel, liposomes layered with PLL and HA. Right panel, magnification of layers ×4. (c) The hydrodynamic size of the nanoparticles increased by approximately 10 nm per layer. (d) The zeta potential confirms the transformation of the negatively charged (−41 ± 8 mV) liposomes surface to positive (31 ± 6 mV) upon layering of the polycation PLL, followed by net charge reversal to (−27 ± 8 mV) upon deposition of the polyanion HA. (e) The polydispersity index (PDI) revealed that the overall size of the nanoparticles remained homogeneous, with no second‐degree aggregation formation during formulation. Combination release of (f) AZD2281 and cisplatin, (g) BMN 673 and cisplatin from the polymeric nanoparticles at 37°C in an excess volume of buffered citrate PBS in a time‐dependent manner at pH 5.0 (**p < .001, ***p < .0001) than at pH 5.0. (h) release of cisplatin from polymeric nanoparticles at 37°C in an excess volume of PBS at pH 5.0 and pH 7.4 (***p < 0.0001)

We designed single modular NPs for the efficient encapsulation of cisplatin and PARPi for synergistic dual‐drug delivery (Figure 1a). Two PARPis were used: AZD2281 (olaparib), which is FDA approved for germline *BRCA*‐mutated (gBRCAm) advanced ovarian cancer,^15^ and BMN 673, which is currently in clinical trials for *BRCA*‐deficient ovarian cancer patients.^28^ Both AZD2281 and BMN 673 are very hydrophobic, with poor solubility of 0.1 mg/mL in water.^14^

We successfully formulated liposomal NPs by self‐assembly of the lipids DSPC (1,2‐distearoyl‐*sn*‐glycero‐3‐phosphocholine, POPG (1‐palmitoyl‐2‐oleoyl‐*sn*‐glycero‐3‐phospho‐(1′‐*rac*‐glycerol)) (sodium salt), and cholesterol in a mass ratio of 56:39:5. The PARPis were introduced in a chloroform–ethanol mixture during liposome preparation. Due to their hydrophobicity, the PARPis partitioned into the liposomal bilayer (Figure 1a). Cholesterol was added to stabilize and compact the liposomes. The liposome film was hydrated under sonication with 300 mM citric acid solution (pH 4). Cisplatin was dissolved in 0.9% sodium chloride solution (1 mg/mL; pH 7.4) under sonication at 65°C for 10 min to ensure complete drug dissolution prior to addition to the liposomal suspension under sonication for 15–30 min. The encapsulated NPs were washed with tangential flow filtration (TFF), and the amount of loaded cisplatin was determined using inductively coupled plasma mass spectrometry (ICP‐MS). The encapsulation efficiency of cisplatin was 64%, with a drug/lipid ratio of 9.7% (w/w). The net encapsulation efficiency was 26% for AZD2281, corresponding to a drug/lipid ratio of 2.5% (w/w), and 21% for BMN 673, corresponding to a drug/lipid ratio of 2.4% (w/w), as determined by high performance liquid chromatograph mass spectrometry (HPLC/MS). The free drugs were initially purified by filtration through a sterile 0.2‐μm filter membrane, followed by TFF. Final liposome purification and concentration were performed using TFF.^41^

To enhance the structural stability, cell‐targeting capability, and dual‐drug release mechanism of the NP platform, we employed an LbL polyelectrolyte deposition approach.^32^, ^36^ Polycation PLL (15–30 kDa) was deposited on the negatively charged liposomes to provide stability and pH sensitivity, followed by deposition of the polyanion, hyaluronic acid (HA, 40 kDa), to form the terminal layer. HA is a ligand for the receptor CD44, which is highly over‐expressed on most ovarian cancer cells,^42^, ^43^ and hence functions as a targeting layer on the NPs (Figure 1a). Cryo‐transmission electron microscopy (TEM) (TEM; Figure 1b) revealed that the diameters of the NPs were consistent with the average liposome hydrodynamic size (*z*‐average) of 90 ± 12 nm measured by dynamic light scattering (DLS) (Figure 1c). The liposome diameter increased by ~10 nm upon sequential deposition of polyelectrolyte layers of PLL and HA (Figure 1b,c). The successful sequential deposition of these polyelectrolytes was further confirmed by the charge reversal of the initial zeta (ζ) potential of the surface charge of the liposomes from −43 ± 5 to 26 ± 5 mV upon PLL addition, followed by reversal to a final net surface charge of −31 ± 6 upon deposition of the polyanion HA (Figure 1d). The final particles exhibited a polydispersity index (PDI) of 0.12 ± 0.02 (Figure 1e).

The dual‐drug release kinetics of the LbL NPs were investigated at 37°C in 300 mM citrate buffer with PBS at pH 5.5 to mimic physiological conditions in the endosomal compartment and tumor microenvironment.^44^ As expected, AZD2281 (Figure 1f) and BMN 673 (Figure 1g), small hydrophobic molecules entrapped by the phospholipid bilayer of the liposomes, were released first, followed by cisplatin. We hypothesized that this release profile would allow the PARPis to sensitize HGSOC cells by downregulating PARP protein activity and induce SSBs^12^, ^45^ prior to cisplatin release to potently induce DSBs.^40^, ^46^

### 2.2 Encapsulated PARPi and cisplatin NPs inhibit DNA repair via HR

**Figure 2:**
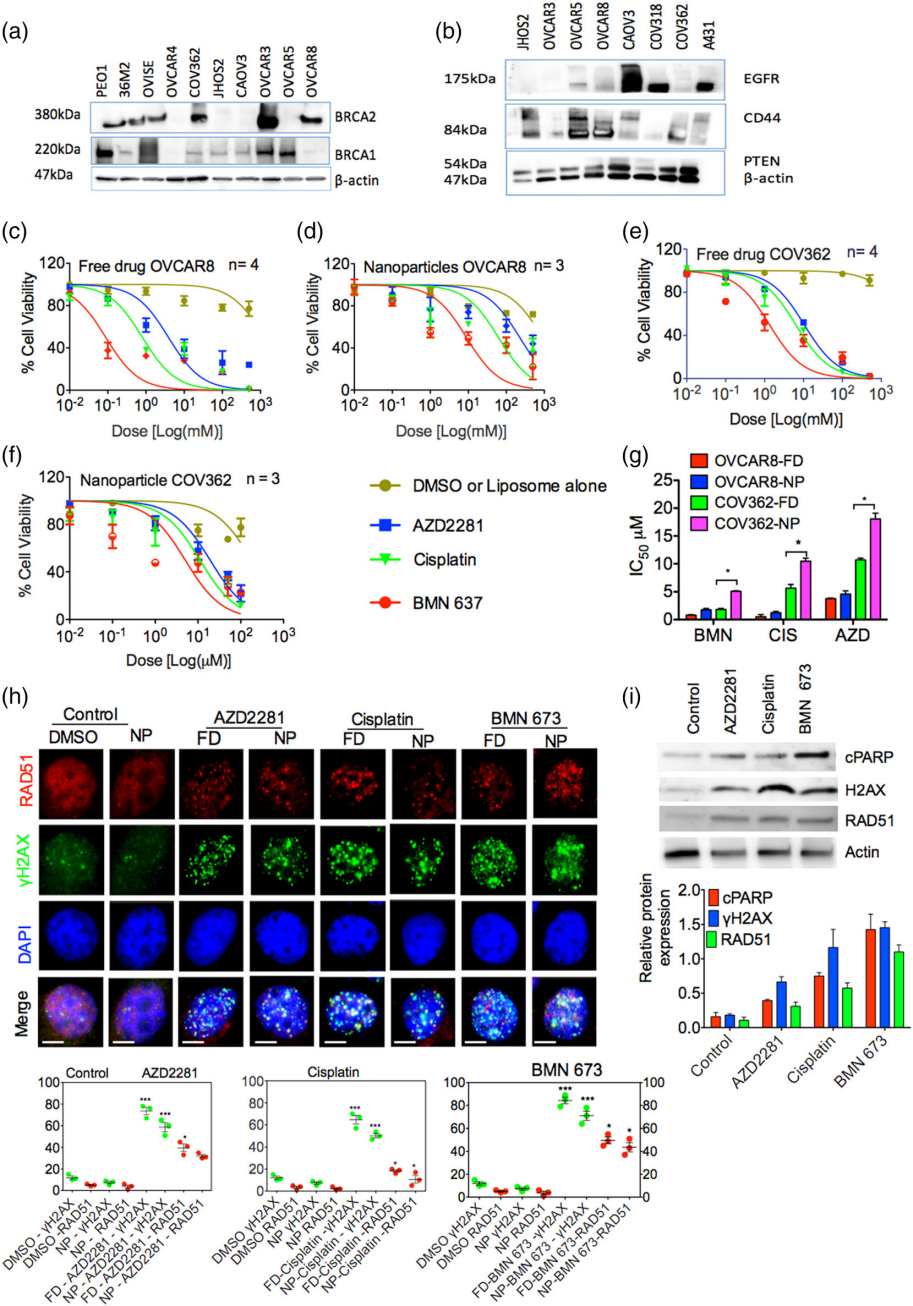
PARPi and cisplatin induce apoptosis via DNA damage and inhibition of homologous recombination. Western blot showing (a) *p53* and *BRCA* mutation and (b) CD44 expression status in A431 cells and a cohort of ovarian cancer cell lines. Dose–response curves for OVCAR8 cells treated with (c) free drug (FD) and (d) encapsulated nanoparticles (NPs) AZD2281, BMN 673, and cisplatin. Dose–response curves for COV362 cells treated with (e) free and (f) encapsulated AZD2281, BMN 673, and cisplatin. (g) IC_50_ of the OVCAR8 and COV362 dose–response curves. (h) Detection (top panel) and quantification (bottom panel) of RAD51, γH2AX foci formation, and 4′,6‐diamidino‐2‐phenylindole (DAPI) by immunostaining in OVCAR8 cells after 24 hr of treatment with AZD2281, cisplatin, and BMN 673. I, Western blot showing relative expression (top panel) and quantification (bottom panel) of cleaved PARP (cPARP), RAD51, and γH2AX in OVCAR8 cells after 24 hr of treatment with 1 μM of AZD2281, cisplatin, and BMN 673. The data represent at least three independent experiments and are presented as the mean ± *SEM*. Statistical analysis was performed by one‐way ANOVA; *p < .05, **p < .01, ***p < .001. PARPi, poly(ADP‐ribose) polymerase inhibitors

After evaluating *BRCA1/2* and *p53* mutation status and CD44 expression levels in HGSOC and other ovarian cancer cell lines by western blot (Figure 2a,b), we elected to further study OVCAR8 and COV362, which carry due to promoter hyper methylation and *BRCA1* mutations^47^ and have high and moderate CD44 expression levels, respectively. These cell lines are of interest to us because of the CD44‐targeting ability of our HA‐coated LbL NPs. Cell viability assays were performed to assess the efficacy of the free and encapsulated drugs in OVCAR8 cells (Figure 2c,d) and COV362 cells (Figure 2e,f) and to determine their apparent IC50s (Figure 2g). Previous preclinical studies have shown that BMN 673 is significantly more potent than other PARPis,^28^, ^48^ and in our cell viability assay (Figure 2g) we observed a significant difference (*p* < .05) in potency between BMN 673 and AZD2281. Interestingly, BMN 673 was also significantly more potent than cisplatin (*p* < .05) based on IC50 values, consistent with the literature.^28^, ^48^ Notably, OVCAR8 cells were more sensitive than COV362 cells to PARPi treatment, and similar dose‐dependent cytotoxicity was observed in OVCAR4, Kuramochi, and OVISE cells (Figure S1a–c). We believe that the effectiveness of the BMN 673‐containing liposomes is due to its broad targeting and tight inhibition of PARP1 catalytic activity, as reported by Shen et al.^28^ and Murai et al.^48^

To determine if the cytotoxicity observed in the cell viability assays was due to induction of apoptosis as a result of inhibition of DNA repair, OVCAR8 and COV362 cells were plated and incubated with ~10 μM free or NP‐encapsulated AZD2281, BMN 673, or cisplatin for 24 hr. The cells were then stained for RAD51, an indicator of HR during DNA break and phosphorylated γH2AX(Ser139), a surrogate marker of induction of the DNA damage response^49^ (Figure 2h). γH2AX foci formation was significantly increased in drug‐treated cells compared to cells treated with dimethyl sulfoxide (DMSO) or blank liposomes (*p* > .001), confirming induction of DNA damage and inhibition of double‐strand DNA repair.^50^ Significant differences were observed between the various drug treatment groups. Interestingly, RAD51 and γH2AX(ser139) foci formation levels were higher in OVCAR8 cells than in COV362 cells (*p* < .001), indicating greater sensitivity of OVCAR8 due to *BRCA1* promoter hypermethylation (Figure 2h and Figure S1d). This observation is consistent with the status of *BRCA1* in these cell lines. OVCAR8 cells carry residual *BRCA1* expression, whereas COV362 carry *BRCA1* mutation.^47^, ^51^ Overall, in both cell lines, the free drugs showed greater potency than the encapsulated drugs, most likely due to the faster delivery of the free drugs to cells; the encapsulated drugs require an initial step of CD44 receptor‐mediated endocytosis of the LbL NPs as well as controlled release from the carrier. Finally, these findings are also consistent with the significant elevation of γH2AX(Ser139) expression compared to RAD51 in *BRCA*‐mutated cell lines after exposure to DNA‐damaging agents.^49^, ^52^

In addition to their role in DNA repair, PARPs are also substrates for Caspase 3 cleavage to initiate apoptosis. During apoptosis, PARP proteins undergo proteolytic cleavage, which can be monitored by western blot. The main PARP protein cleavage site is Asp214/Gly215, and the two resulting protein fragments can be used as markers of cleaved Caspase 3‐mediated apoptosis activation.^53^ We therefore investigated the presence of apoptosis in OVCAR8 cells by incubating the cells with ~10 μM encapsulated AZD2281, BMN 673, or cisplatin for 24 hr. Compared to untreated cells, we observed elevated levels of cleaved PARP (Asp214) proteins by western blot. These results are consistent with the increased expression of γH2AX(Ser139) and RAD51. Cleaved PARP levels were highest in BMN 673‐treated cells, consistent with the higher potency of BMN 673 compared to other PARPis in our cell viability assays and other reports^29^, ^48^ (Figure 2i).

### 2.3 LbL polymeric liposomal NPs exhibit a prolonged circulation half‐life and selective in vivo targeting of serous ovarian cancer cells

**Figure 3:**
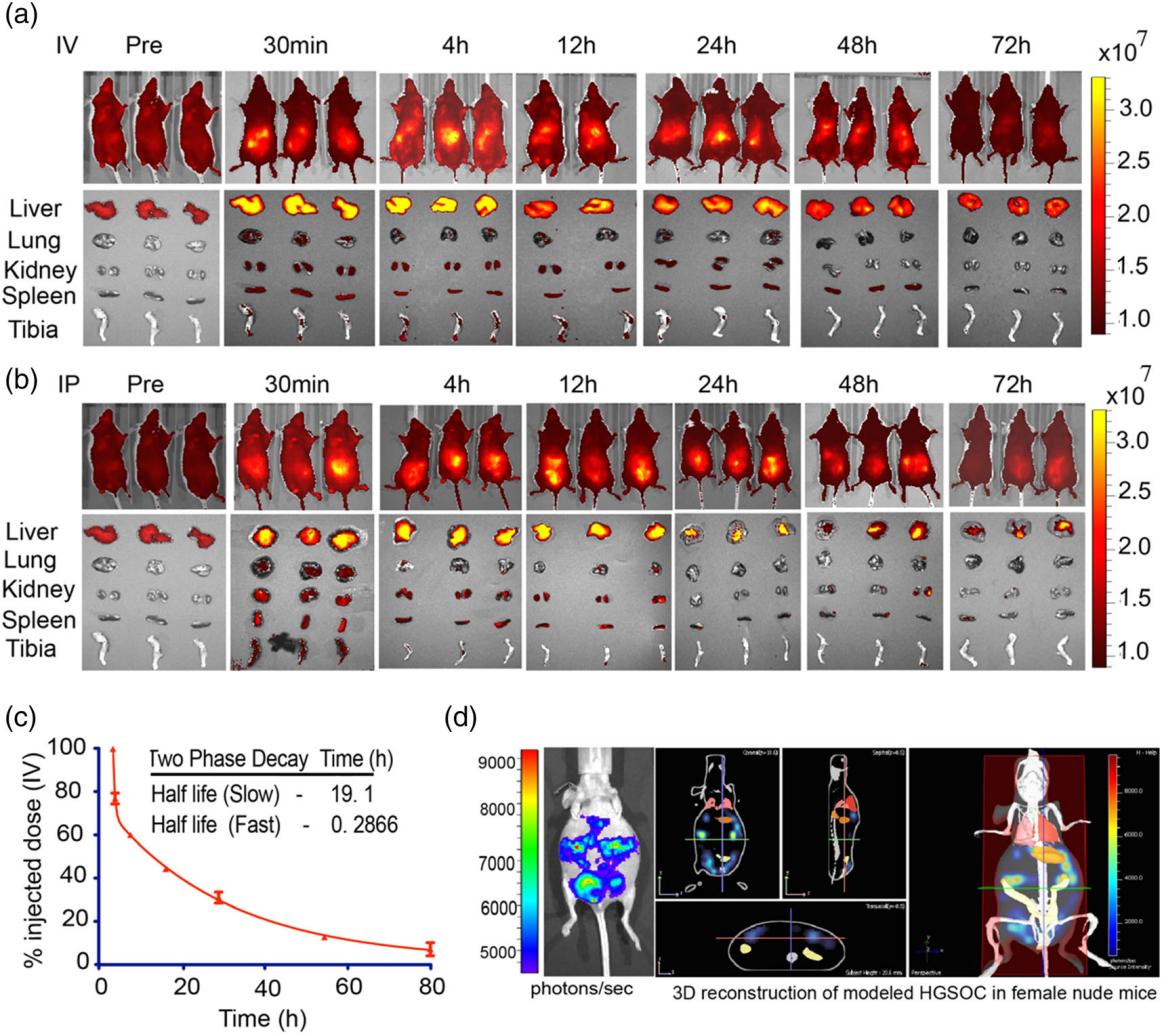
Polymeric liposomal nanoparticles with a terminal HA layer exhibit prolonged blood circulation and selective targeting of CD44 on COV362 cells. Biodistribution of blank Cy5.5‐labeled polymeric liposomal nanoparticles in immunocompetent BALB/c female mice and vital organs after (a) intravenous (IV) and (b) intraperitoneal (IP) injection. (c) The Cy5.5 fluorescence signal was quantified in retro‐orbital blood samples taken at specific time intervals, and the percentage remaining dose was calculated based on the initial injection dose. The nanoparticle half‐life decay was calculated using the two‐compartmental model. Data are presented as the mean ± *SEM*, *n* = 3 mice per time point. (d) Modeling of orthotopic high‐grade serous ovarian cancer (HGSOC) in NCR nude female mice with luciferase‐ and CD44‐expressing OVCAR8 xenografts. The disease phenotype was acquired with an International Veterinary Information Service (IVIS) bioluminescence imager (left panel), tumor metastasis is presented as 3D tomography in the sagittal, coronal, and transverse planes (middle panel), and whole body (right panel). HA, hyaluronic acid

Successful in vivo therapeutic delivery and tumor targeting require a nanoscale carrier with robust architectural stability and stealth‐like capability to avoid degradation, rapid systemic clearance, and sequestration of NPs in vital organs.^33^, ^54^, ^55^ We investigated the pharmacokinetics and biodistribution profile of our new LbL NPs by intravenous (IV) or intraperitoneal (IP) administration of 100 μL (2.5 mg/kg) of blank PLL‐Cy5.5/HA‐labeled liposomal NPs to two cohorts of 4‐ to 6‐week‐old healthy immunocompetent BALB/c female mice. Blood samples were drawn at pre‐injection and at 30 min, 4 hr, 12 hr, 24 hr, 48 hr, and 72 hr. Whole‐body bioluminescence imaging signals and microplate readings of the Cy5.5 absorbance of the blood samples were used to calculate the percentage remaining of the NP dose from the time points pre‐ and post‐NP injection. Compared to IP administration, IV administration produced a significantly lower nonspecific tissue/organ NP biodistribution. At 30 min, 4 hr, 24 hr, and 72 hr after IV administration, 48, 51, 38, and 9% of the Cy5.5 fluorescence signal of the NPs remained (Figure 3a), compared to 42, 47, 39, and 13%, respectively, for IP administration (Figure 3b). Similar time‐dependent decreases in organ NP accumulation were observed in the liver, kidney, and spleen (Figure 3a,b). These observations support the favorable clearance and biodistribution profile of the NPs. According to the two‐compartment model,^56^ the systemic blood circulation half‐life of the NPs following IV injection was 0.29 hr for fast decay and 19.1 hr for slow decay (Figure 3c). These data indicate that the NP‐mediated drug delivery provided prolonged bioavailability in mice, with a half‐life of 19.1 hr. This observation is consistent with bioavailability of PARPi in vivo when delivered by the conventional approach, as shown by data from Rothenberg et al. on rapid plasma clearance of AZD2281 in tumor‐bearing mice.

Hyaluronic acid‐terminated NP particles have previously been show both in vitro and in vivo to successfully colocalize with CD44 expression on ovarian cancer cells.^57^, ^58^, ^59^ These studies demonstrate that HA‐terminated NPs will successfully target and bind CD44 receptors on ovarian cancer cells such as OVCAR8, which expresses high levels of the receptor. OVCAR8 cells have previously been shown to form uniform tumor size and proliferate linearly in vivo.^60^ To validate work done by Mitra et al. 2015 in our laboratory setting, we performed preliminary studies to investigate the tumor formation and growth kinetics of COV362, OVCAR8, OVCAR4, and Kuramochi HGSOC cells in NCR female nude mice. The OVCAR8 cells tumor growth observed were consistent with work done by Mitra et al. 2015. We therefore selected OVCAR8‐luciferase expressing cells to model orthotopic HGSOC pathology in female nude mice. This disease model was established by IP implantation of OVCAR8 cells adjacent to rather than in the ovaries. The characteristics of HGSOC include the presence of aggressive tumor nodules and disseminated micrometastases, particularly in the peritoneal cavity and often near vital organs such as the liver, lungs, and spleen. Injection of firefly luciferin followed by bioluminescence imaging and whole‐body 3D reconstruction revealed an orthotopic xenograft ovarian cancer tumor phenotype in vivo recapitulating that of HGSOC pathology (Figure 3d).

### 2.4 Evaluation of the hematological toxicity of NPs versus free drug delivery in vivo

Overall, in vivo preclinical testing and clinical trials have shown that the significant treatment response of cisplatin and PARPI combination therapy is accompanied by intolerable overlapping systemic toxicity, which requires a significant reduction of the dosing of either cisplatin or PARPI or their combination. Here, we administered escalating doses of cisplatin, AZD2281, and BMN 673 as monotherapies or combination therapies.^61^ Two different dose studies were performed. In the first dose study, the mice received the selected dose of each drug or the combination for three consecutive days, followed by thrice‐daily monitoring for 2 weeks.^61^, ^62^ In the second dose study, the mice received only a single dose and were again monitored for a 2‐week period. In both studies, 6‐ to 8‐week‐old healthy immunocompetent BALB/c female mice were dosed with cisplatin, AZD2281, or BMN 673 individually or in combination as free drugs or encapsulated in NPs using the dosages previously described with moderate adjustment.^28^ The standard procedure used is to determine the core NP drug dosing based on the cisplatin concentration.

**Figure 4:**
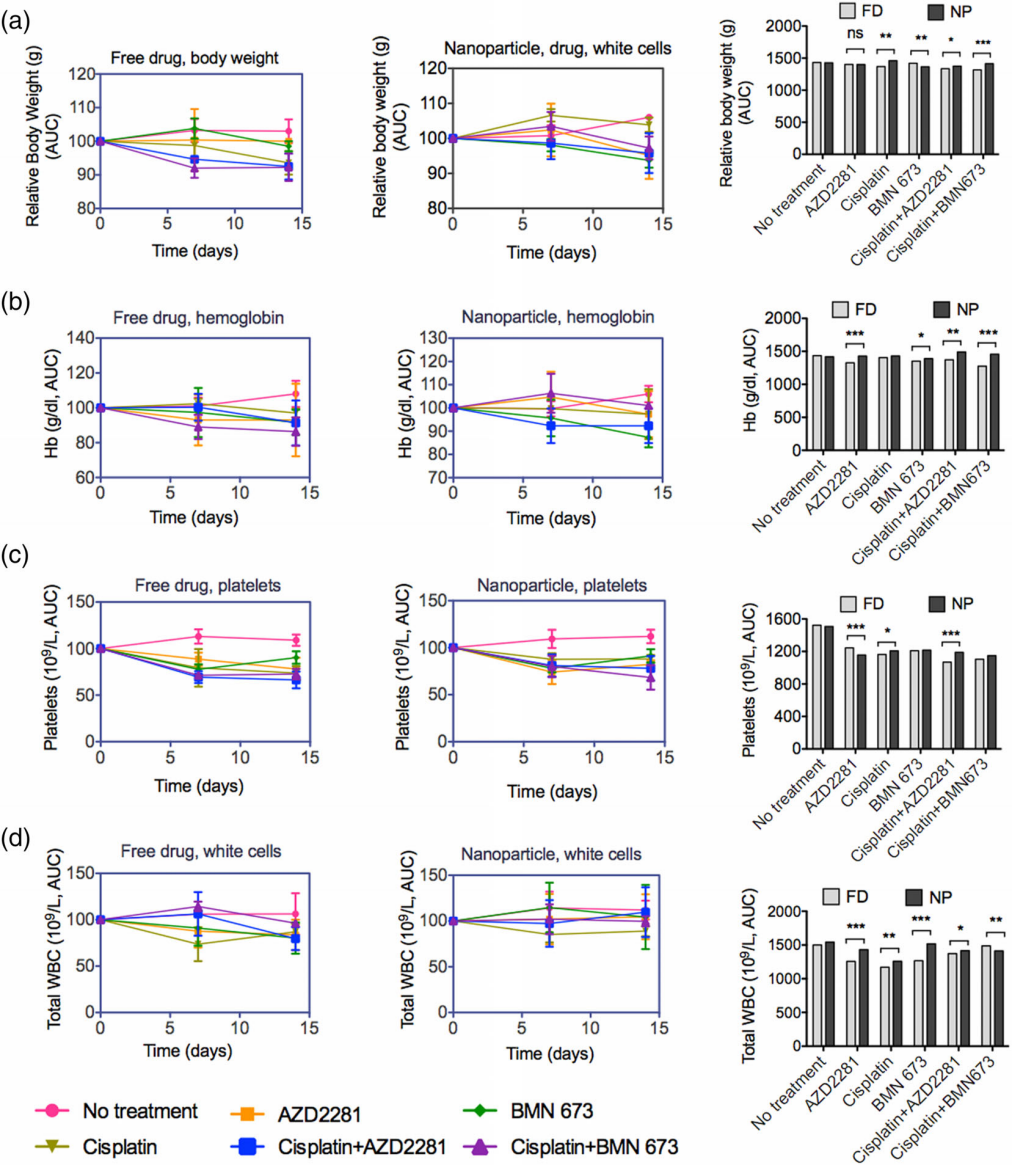
Maximum‐tolerated drug dose (MTD) studies revealed that encapsulated delivery was better tolerated than the free drugs. (a) Body weight, (b) hemoglobin (Hb), (c) platelets, and (d) total white blood cells (WBC) were measured 2 weeks after IV injection of NCR nude female mice with single monotherapy of AZD2281, BMN 673, or cisplatin or cisplatin combined with AZD2881 or BMN 673 in free drug (FD, left panel) or nanoparticle‐encapsulated form (NP, right panel). The data were normalized to untreated mice, analyzed as the area under the curve (AUC) and plotted as histograms. Data are presented as the mean ± *SEM*, *n* = 3. Statistical analysis was performed by one‐way ANOVA; *p < 0.05, **p < 0.01, ***p < 0.001. IV, intravenous

In the first high‐dose study (Figure S2a), mice administered only cisplatin, AZD2281, or BMN 673 in free form exhibited severe body weight loss (>10% of original body weight) (Figure S2a; *p* < .0002), anemia (Figure S2b), and pancytopenia (Figure S2d; *p <* .00081). In addition, severe loss of body weight and pancytopenia were observed in mice treated with free cisplatin in combination with AZD2281 or BMN 673 on day 6 postdrug administration. In the cohorts of mice treated with free cisplatin combined with AZD2281 or BMN 673, two and three of the five mice in each group, respectively, died 8–10 days postdrug injection. Among the mice treated with the encapsulated drugs, no mortality was observed, and mice treated with only cisplatin, AZD2281, or BMN 673 or cisplatin combined with AZD2281 or BMN 673 also exhibited appreciable body weight loss or pancytopenia (Figure S2a), which could be an indication of dose limitation in the NP drug combination. In the second (single dose) study, moderate (5–10% of body weight) body weight loss and myelosuppression were observed in mice injected with free cisplatin, AZD2281, or BMN 673 only, with moderate to severe effects in mice injected with cisplatin in combination with AZD2281 or BMN 673. By contrast, mice injected with the encapsulated drugs exhibited only mild (<5% of original count) pancytopenia (AZD2281, *p* < .00065; cisplatin, *p* < .0067; BMN 673, *p* < .00023; Figure 4d) and mild body weight loss (<5% of original body weight) over the course of the 2‐week study period (Figure 4a). The findings for the free drug groups are consistent with published preclinical data. Interestingly, the mice treated with LbL NPs exhibited significantly reduced hematological toxicity, indicating the increased safety of LbL NP drug delivery for combination therapy compared to the conventional approach.

### 2.5 Codelivery of encapsulated cisplatin and PARPi significantly regresses tumor growth in vivo in orthotopic HGSOC xenografts

Six‐ to eight‐week‐old female Crl:Nu (NCR) nude mice bearing tumors after orthotopic implantation with luciferase‐expressing OVCAR8 cells were used to assess therapeutic efficacy in tumor regression. The mice were randomly allocated to treatment groups and dosed weekly with free or encapsulated AZD2281, BMN 673, or cisplatin or cisplatin combined with AZD2281 or BMN 673. Tumor regression was monitored weekly by whole‐body bioluminescence imaging after injection with firefly luciferin. The treatment response was evaluated based on the tumor bioluminescence signal, Kaplan–Meier survival plots and body weight and compared between the different treatment groups.

**Figure 5:**
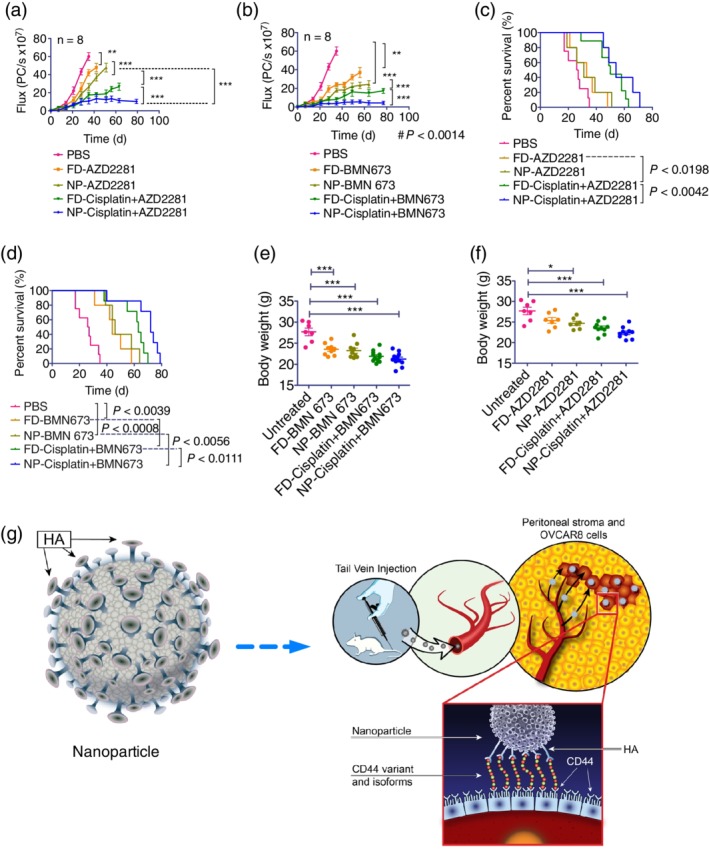
HA terminal‐layered polymeric liposomal nanoparticles produce a superior in vivo therapeutic response in HGSOC xenografts. NCR nude female mice bearing luciferase‐ and CD44‐expressing OVCAR8 xenografts were treated weekly via IV administration of vehicle, AZD2281, BMN 673, or cisplatin monotherapies or cisplatin combined with AZD2281 or BMN 673 (*n* = 8). (a and b) Plots of the bioluminescent signal flux of the tumors from the start of treatment. Statistical significance was determined by one‐way ANOVA with Turkey's multiple comparison tests. (c and d) Kaplan–Meier plots of survival fractions. Statistical significance was determined using the log‐rank (Mantel–Cox) test. (e and f) The body weight distribution of the treatment groups was measured and graphed as scatter plots. Statistical significance was determined by one‐way ANOVA with Bonferroni's multiple comparison tests. Data are presented as the mean ± *SEM*; *p < .05, **p < .01, ***p < .001. (g) Schematic illustration of the design of the polymeric liposomal nanoparticle assembly with loaded therapeutic cargo and the treatment mechanism. FD, denote free and NP‐encapsulated nanoparticles. HGSOC, high‐grade serous ovarian cancer; IV, intravenous

There was a significant improvement in therapeutic response for monotherapy compared with PBS in the cohort of mice treated with free cisplatin (*p* < .0026), AZD2281 (*p* < .0016), or BMN 673 (*p* < .0039) and for encapsulated monotherapy compared with liposome vehicle alone in the cohorts of mice treated with cisplatin (*p* < .0001), AZD2281 (*p* < .0014), or BMN 673 (*p* < .0008). Similarly, a significant improvement in the treatment response was observed in mice treated with free or encapsulated cisplatin monotherapy versus combination therapy with BMN 673 (*p* < .0038). Similar levels of effectiveness were observed for free cisplatin monotherapy compared with cisplatin combined with either AZD2281 or BMN 673 (*p* < .0002), for encapsulated AZD2281 compared with encapsulated cisplatin combined with AZD2281 (*p* < .0198), and for encapsulated BMN 673 compared with encapsulated cisplatin combined with BMN 673 (*p* < .0056). Notably, the combination of cisplatin with BMN 673 or AZD2281 was more effective in encapsulated form than in free form (*p* < .0111). Among all treatment groups, encapsulated cisplatin combined with BMN 673 produced the greatest improvement in the treatment response (Figure 5a,b and Figure S4a,b). In conclusion, the encapsulated single‐ and dual‐drug therapies reduced tumor burden and metastasis more effectively over time than the corresponding free drug versions. The greater therapeutic efficacy of the encapsulated drugs is presumably attributable to the selective targeting of CD44 on OVCAR8 cells by HA (Figure 5f and Figure S4f). The proposed mechanism of HA‐terminated interaction with HGSOC is illustrated in Figure 5g.

The Kaplan–Meier plots of the survival fractions as a function of treatment response were consistent with the bioluminescence treatment response data. We observed statistically significant differences (*p* < .0002) between all untreated and treated survival times. Treatment with encapsulated cisplatin combined with BMN 673 increased survival to 79 days, compared to 70 days for treatment with the free drugs. Similarly, treatment with encapsulated cisplatin or AZD2281 increased survival to 71 days, compared to 63 days for the corresponding free drug treatments (*p* < .0002). Treatment with encapsulated BMN 673 increased survival to 64 days, compared to 58 days for the free drug treatment (*p* < .0002) (Figure 5c,d and Figure S4c,d). Overall, compared with free drugs, treatment with encapsulated drugs improved survival by 10.34–12.85% among the various treatment groups.

In untreated mice, tumors grew rapidly, and the mice were sacrificed at 35 days post‐tumor implantation (Figure 5e,f and Figure S3e,f). The body weights of the mice treated with vehicle alone increased rapidly, consistent with the overall increase in tumor burden and the increased volume of ascites. By contrast, the body weights of mice treated with free or encapsulated monotherapies were relatively stable, and the differences in body weight between the untreated controls and the mice receiving the free or encapsulated drug combinations were statistically significant (*p* < .001). The difference in body weight between the mice treated with liposome vehicle alone and with the encapsulated combination therapies was also significant (*p* < .0001; Figure 5e and Figure S3e).

**Figure 6:**
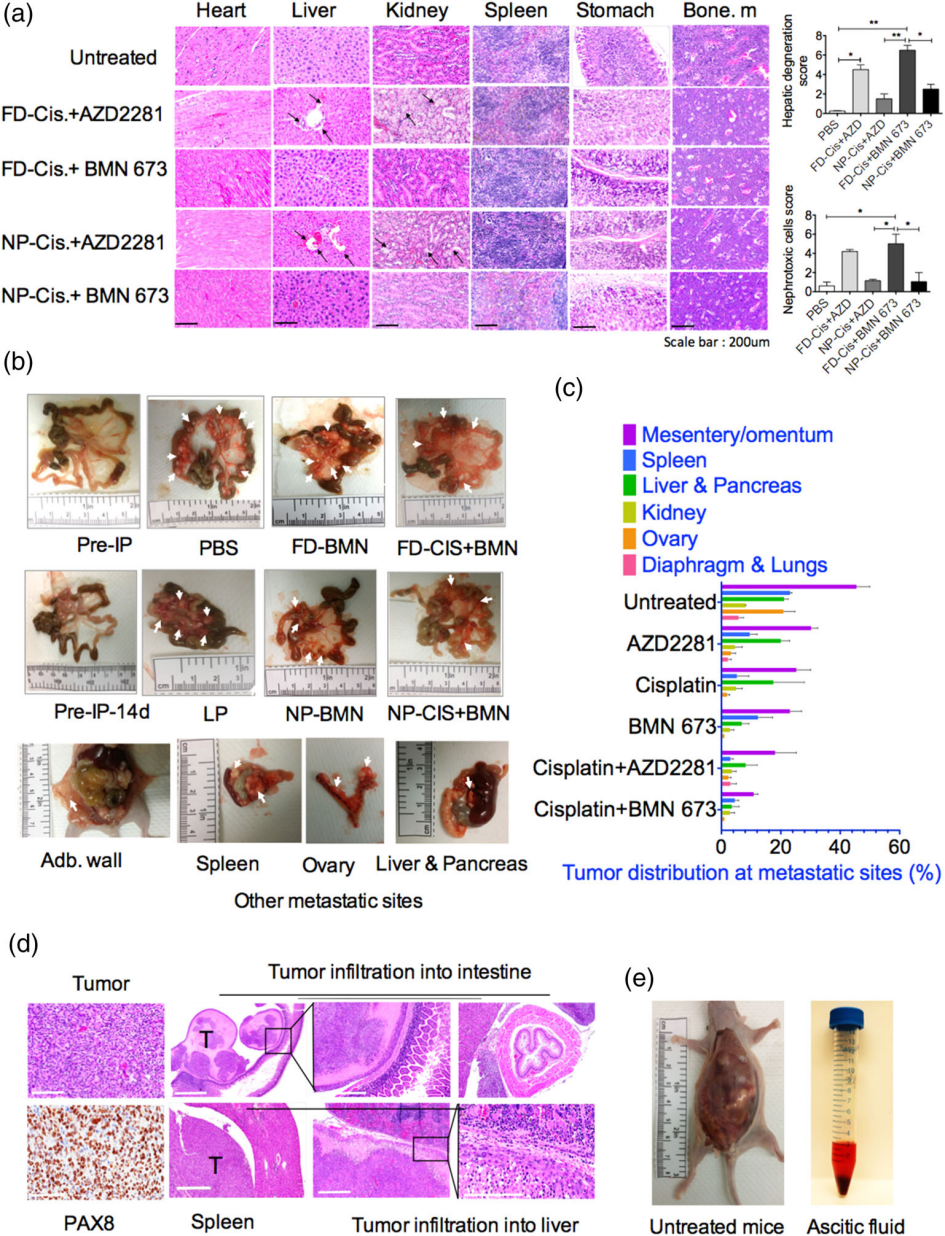
Nanoparticle‐treated xenograft mice exhibit reduced organ toxicity and diminished tumor burden. (a), Histological analysis by hematoxylin and eosin (H&E) staining of the organs of groups receiving cisplatin combined with AZD2281 and/or BMN 673 compared with untreated mice and quantification of hepatic degeneration and nephrotoxic cells. (b) Light micrographs of freshly excised mice mesentery showing OVCAR8 tumor burden and other vital organs in mice treated with free or encapsulated BMN 673 alone or combined with cisplatin. (c) Bar plots of OVCAR8‐tumor metastases at different anatomic sites of peritoneal organs, including the mesentery/omentum, spleen, liver, pancreas, kidney, ovary, diaphragm, and lungs in the nanoparticle‐treated groups. (d) Immunohistochemical analysis of OVCAR8 tumor tissue by PAX8 and H&E staining showing OVCAR8 tumor cell infiltrations into the liver, spleen, and intestinal tissues. (e) Control and OVCAR8 xenograft‐bearing NCR nude female mice and extracted peritoneal fluids showing ascites in the untreated mice. FD, free drug; NP, nanoparticle

The variation in body weight of mice can pose a challenge to its use as the sole indicator of toxicity in in vivo studies. We therefore performed H&E staining to analyze the vital organs of the cohort of mice that received the dual‐combination treatment. Tissue histology revealed that the mice treated with free cisplatin and AZD2281 exhibited high numbers of white pulp cells of lymphoid origin in the spleen and significant numbers of nucleated red blood cells in the bone marrow. In mice treated with free cisplatin and BMN 673, large, immature, and enlarged hepatocytes were observed; in the kidney, there were enlarged tubules with necrotic cells and fewer nephrons, possibly due to tubular damage. These observations are suggestive of liver and kidney atrophy with accompanying myelodysplastic features in the bone marrow of these mice. By contrast, mice treated with encapsulated cisplatin and AZD2281 and BMN 673 displayed less liver and kidney toxicity compared to mice treated with the drugs in free form. Close microscopic examination and scoring revealed the presence of 1.15 and 1.0 nephrotoxic cells per field in encapsulated cisplatin‐AZD2281 and cisplatin‐BMN 673 treated animals, respectively, compared to 4.2 and 5 nephrotoxic cells per field in cisplatin‐AZD2281 and cisplatin‐BMN 673 free‐drug treated groups, respectively (Figure 6a), which was statistically significant (*p* < .0139). Similarly, severe to mild hepatic degeneration was observed in mice treated with free cisplatin and BMN 673 which contains 6.5 nephrotoxic cells per field compared to 2.5 nephrotoxic cells in mice treated with the encapsulated cisplatin and BMN 673 drugs (*p* < .0217; Figure 6a). Nucleated red blood cells and immature myeloid cells in the bone marrow were also markedly increased 30% in both cisplatin combinations with either AZD2281 or BMN 673 free drug‐treated mice compared to the NP‐treated group tissues (Figure 6a). Thus, the nanocarriers significantly reduced or eliminated the systemic toxicity associated with the delivery of these drugs via the conventional free drug approach.

Ovarian cancer is thought to originate from mesenchymal stem cells of the ovaries^63^ and fallopian tubes,^64^ and the mesentery and its surrounding blood vessels and lymph nodes are favorable metastatic sites during ovarian cancer cell proliferation. Assessment of the level of metastasis within the peritoneum in the treated mice revealed a correlation between the treatment response and the level of OVCAR8 cell tumor burden within the mesentery and surrounding organs (Figure 6b). Furthermore, there were significant reductions in tumor burden at various metastatic sites in the cohort of mice treated with encapsulated cisplatin and BMN 673 compared with those treated with encapsulated cisplatin and AZD2281, the corresponding monotherapies, and the no treatment group (Figure 6b,c). We also performed immunohistochemical staining of tumor cells for the Paired‐box gene 8 (PAX8) protein, which is used as a differential marker of EOC.^65^ H&E staining was performed to examine and confirm tumor infiltration into key organs such as the intestine, spleen, and liver (Figure 6d). The increase in volume of peritoneal ascites also correlated with the mesenteric tumor burden across most of the treatment groups. Mice treated with PBS were used as a positive control, and tumor‐free mice were used as a negative control without ascites (Figure 6e).

### 2.6 Correlation of apoptotic markers and biochemical metabolites with treatment response

**Figure 7:**
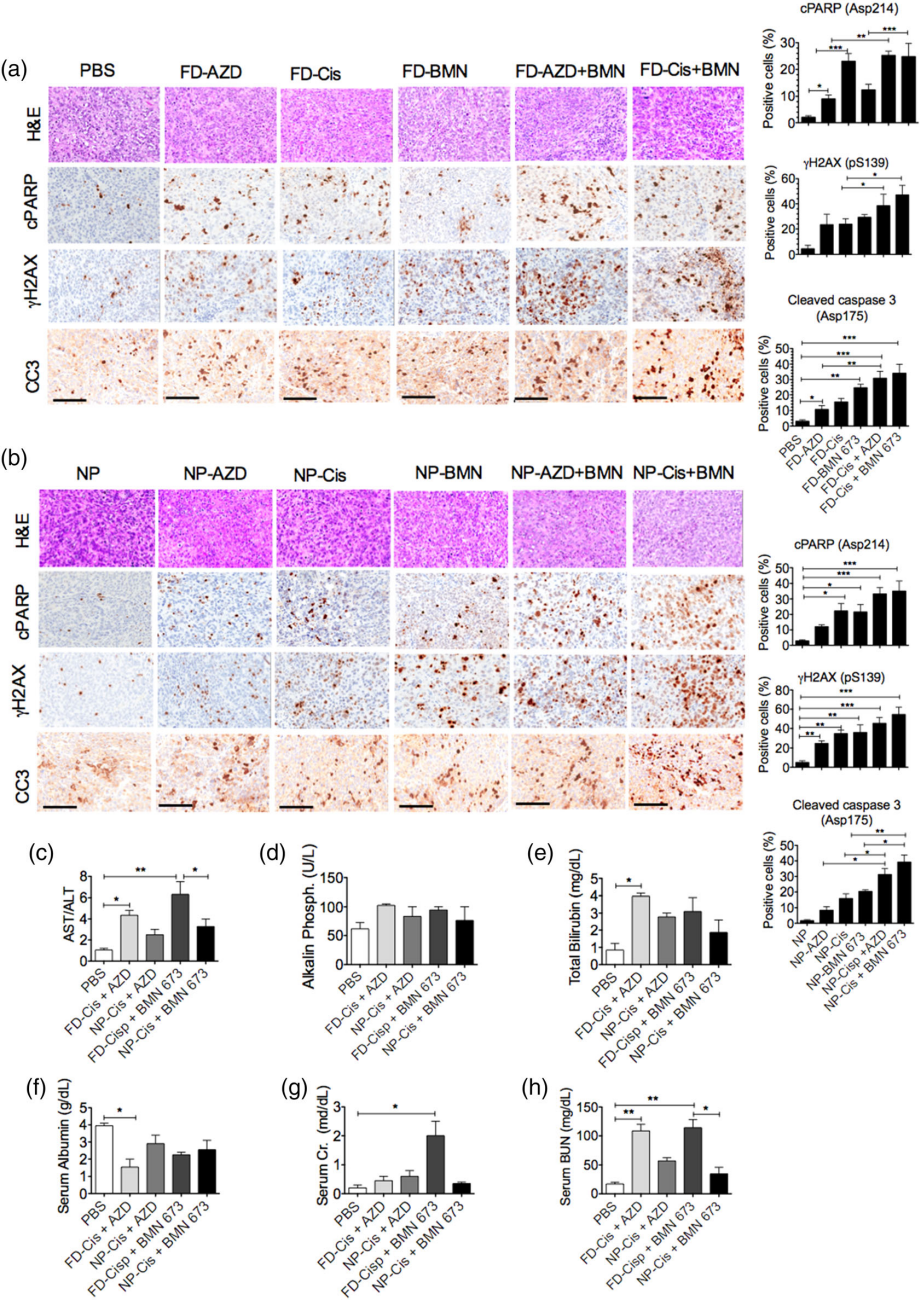
Encapsulated dual delivery of PARPi and cisplatin significantly inhibits homologous recombination, elevates apoptotic markers, and decreases systemic toxicity in xenograft‐bearing mice. Top panel, H&E; remaining panels, immunohistochemical analysis and quantification of cleaved PARP (cPARP), γH2AX(pSer139) foci formation, and cleaved Caspase 3(CC3) expression in tumors treated with (a), free drug (FD) or (B) nanoparticle (NP)‐encapsulated AZD2281, cisplatin, BMN 673, dual cisplatin‐AZD2281, or dual cisplatin‐BMN 673. PBS or blank NPs were used as controls, respectively. The graphs present the mean ± *SEM* of the number of positive cells in at least two mouse tumor samples. Statistical significance was determined by one‐way ANOVA with Bonferroni's multiple comparison tests; *p < .05, **p < .01, ***p < .001. Serum blood samples were analyzed for liver function tests, (c) AST/ALT, (d) alkaline phosphatase, and (e) total bilirubin. (f) Serum albumin, (g) serum creatinine (Cr), and (h) serum blood urea and nitrogen (BUN) kidney function tests to assess the levels of organ and systemic toxicity in the treatment groups. The graphs present the mean ± *SEM* of at least two mouse tumor samples. Scale bar: 500 μm for H&E and 200 μm for Immunohistochemistry (IHC). ALT, alanine aminotransferase; AST, aspartate aminotransferase; poly(ADP‐ribose) polymerase inhibitor

The treatment of tumor xenografts with PARPis and cisplatin resulted in tumor regression mediated by apoptotic events induced by the inability of tumor cells to efficiently repair massive DNA damage due to chemotherapy. The induction of apoptosis is marked by increased expression of several proteases that cleave PARPs in response to DNA damage. For example, PI3‐kinases such as ataxia telangiectasia mutated (ATM) rapidly phosphorylate γH2AX(Ser139) at sites of DNA damage to facilitate HR DNA repair. Failure of DNA damage repair initiates Caspase 3‐mediated programed cell death via cleavage of PARP proteins at the site of DSBs. To correlate the level of the therapeutic response with the induction of apoptosis, we stained tumor slices from treated mice for the presence of phosphorylated γH2A.X(Ser139) foci, a surrogate marker of DSBs, cleaved phosphorylated PARP (Asp214) foci, and cleaved Caspase 3 protein expression levels. As anticipated, high levels of γH2AX(Ser139) foci were observed in both encapsulated and free dual drug‐treated tumor tissues compared with single drug‐treated tumors and control untreated tumors. For example, a median of 38.4 and 47 γH2A.X(Ser139) foci were observed cisplatin‐AZD2881 and cisplatin‐BMN 673 free drug‐treated tumor tissues, respectively, compared to 45.2 and 54.2 γH2A.X(Ser139) foci in NP‐treated cisplatin‐AZD2881 and cisplatin‐BMN 673 tissues. While 23. 8 and 29.2 foci were observed in free drug AZD2281 and BMN673 alone treated tissues, respectively (Figure 7a,b). Consistent γH2AX(Ser139) foci elevation in treated tumor tissues, cleaved PARP foci were also markedly increased in tumors treated with dual drugs compared with the single therapies, whether encapsulated or in free drug form, and compared with untreated control tumors (Figure 7a,b). Similarly, high levels of cleaved Caspase 3 staining were observed in the combination treatments compared with the single treatments, again indicating the induction of apoptosis and tumor shrinkage (Figure 7a,b). Various tissues were also used as controls to validate the quality of staining (Figure S3).

We further investigated the association of systemic toxicity with treatment response in mice that showed a significant loss of body weight during treatment. As previously noted, body weight analysis alone is not sufficient for inferring the level of systemic toxicity of chemotherapy. We therefore employed both histochemical and biochemical analyses to evaluate the level of toxicity, particularly in mice treated with drug combinations. A panel of liver and kidney function tests was performed on blood serum samples taken at the time of euthanasia. The serum blood panel includes aminotransferase (aspartate aminotransferase [AST] and alanine aminotransferase [ALT]) levels which are useful biochemical indicators of liver pathology. The AST/ALT ratio revealed mild liver toxicity in mice treated with free cisplatin and moderate to severe liver toxicity in mice treated with free cisplatin combined with either AZD2281 (*p* < .021) or BMN 673 (*p* < .0021) compared to control mice (Figure 7c). By contrast, only mild systemic toxicity was observed in the samples from the cohorts of mice treated with encapsulated cisplatin combined with AZD2281 or BMN 673, consistent with the H&E histology analysis (Figure 7c–f). The AST/ALT ratio in cisplatin and BMN 673 free drug‐treated animals were as high as 6.3 compared to 3.21 in cisplatin and BMN 673 encapsulated treated cohort. Similarly, AST/ALT ratio was 4.37 in cisplatin and AZD2881 free drug‐treated animals versus 2.47 in cisplatin and AZD2881 encapsulated treated group. Similarly, nephrotoxicity was observed in mice treated with free cisplatin combined with either AZD2281 (*p* < .0031) or BMN 673 (*p* < .00219) compared to control mice, as indicated by serum blood urea and nitrogen (BUN) levels (Figure 7g,h). For example, serum BUN level were as high as 114 mg/mL in cisplatin and BMN 673 free drug‐treated animals compared to only 34.5 mg/mL their encapsulated treated cohort. These results suggest a strong therapeutic benefit of LbL NPs as a safe platform for the codelivery of these drug combinations with a limited dosing window due to inherent systemic toxicity.

## 3 DISCUSSION

Extensive preclinical and clinical trial testing of conventionally administered cisplatin and PARPi combination therapies have shown that PARPis are particularly sensitive and effective against ovarian and breast cancers deficient in *BRCA1*, *BRCA2*, and *PTEN* DNA repair genes.^18^, ^62^ The coadministration of PARPis with DNA‐damaging chemotherapies has been extensively shown in clinical trials to prolong tumor‐free survival, improve therapeutic response, and avert the development of resistance due to synergistic effects. However, these clinical benefits are associated with overlapping hematological Grade 3 side effects, and thus full doses of these drug combinations are not tolerated by patients. Development of resistance to cisplatin has been one of the challenges in platinum therapy in clinical settings. Recently several strategies including platinum in combination with antiangiogenic agents such as PARPi have been deployed to achieve success in reversing platinum‐based chemo‐resistant is in many malignancies.^25^, ^26^ These have had more successful in breast and ovarian cancers which lend themselves very well to cisplatin and PARPi combination therapy due to the *BRACness* of these diseases. The advent of targeted nanotherapy also allows for a high dose of cisplatin to be delivered more effectively to cancer cells over healthy cells. Taken together, these findings suggest that the application of targeted delivery of cisplatin and other therapeutics may be sufficient to mitigate platinum resistance particularly in BRCA‐deficient cancers.

In this report, we describe the packaging of cisplatin, AZD2281, or BMN 673 monotherapies and cisplatin in combination with AZD2281 or BMN 673 in an LbL NP drug delivery platform for administration to orthotopic OVCAR8 HGSOC‐xenograft tumor‐bearing mice. This nano‐delivery platform employs liposomes modified with polyelectrolyte nano‐layers to provide improved biodistribution and a terminal functionality that enhances NP trafficking for drug delivery and colocalization into tumors.^36^, ^37^, ^66^, ^67^ The liposomal NPs are layered by electrostatic deposition of a polycation, PLL, to provide structural stability and pH‐responsiveness in the tumor microenvironment, whereas the terminal polyanion layer of the NPs, HA, is critical for selective CD44 targeting of HGSOC tumor cells.^68^ The extravasation of LbL NPs from the blood circulation into the tumor microenvironment (~pH 6.8) facilitates intracellular uptake and trafficking of NPs into the endosomal compartment. The prevailing low endosomal pH of 5.5 enhances the swelling and disassembly of the LbL architecture, resulting in mechanistic release of the PARPi, followed by cisplatin.

This LbL NP drug delivery platform enables ratiometric, synergistic, and modular delivery of combinations of hydrophilic drugs, such as cisplatin, and highly hydrophobic drugs, such as PARPis, that conventional approaches cannot codeliver simultaneously. The mechanistic release of the PARPi followed by cisplatin ensures complete blockade of DNA repair by HR and effective induction of apoptosis, leading to synthetic lethality and a level of effectiveness not achievable by conventional anticancer drug delivery approaches.

A critical attribute of any therapeutic agent is appropriately sustained bioavailability. The LbL NP platform prolongs drug half‐life in the blood circulation and enables tumor cell type‐specific killing while sparing healthy cells, thus avoiding systemic toxicity. Increasing the circulation half‐life of cisplatin simply by increasing the amount of drug is not possible due to the severe side effects associated with its cumulative systemic toxicity, such as myelosuppression, nephrotoxicity, renal dysfunction, and ototoxicity.^25^, ^26^ Myelosuppression, heartburn, abdominal pain, upper respiratory tract infection, and musculoskeletal pain are also associated with conventional delivery of AZD2281 and BMN 673.^69^ These side effects are exacerbated when free cisplatin and PARPi are administered as combination therapy, as observed in our first escalated dose studies. Three mice treated with free monotherapy or cisplatin combined with either BMN 673 or AZD2281 became very moribund, with severe loss of body weight, and were euthanized within 6 days of the study. Hematological analyses at the time of euthanasia revealed pronounced pancytopenia, although we did not perform histological H&E staining for confirmation. By contrast, mice treated with LbL NP‐encapsulated single or dual drugs exhibited only moderate loss of body weight and reduced hemoglobin concentrations compared with untreated mice; the latter exhibited no significant decline in body weight or changes in hematological parameters. Moreover, only mild systemic toxicity was observed in the mice receiving encapsulated dual‐drug therapies, in contrast to the moderate to severe liver toxicity observed in mice receiving the corresponding free forms.

Tumor regression was most significant in mice treated with LbL NP‐encapsulated cisplatin combined with BMN 673, followed by mice treated with encapsulated cisplatin combined with AZD2281, with moderate loss of body weight and overall survival of 80 and 71 days, respectively. By comparison, the level of tumor regression, although significant, was lower in the groups treated with free cisplatin combined with BMN 673 or AZD2281, with overall survival times of 72 and 61 days, respectively. Moreover, in the free drug‐treated groups, there was a significant loss of normalized body weight, most likely due to systemic toxicity. This weight loss was actually countered by an increase in tumor burden and ascitic fluids in the peritoneum. The apoptotic markers phosphorylated (Asp214) cleaved PARP, pH2AX (p139), and cleaved Caspase 3 (Asp175) were significantly increased in tumors treated with free or encapsulated single‐ or dual‐drug therapies compared with untreated tissues.

In summary, we have developed a new, safer nanomedicine LbL NP platform for the effective delivery of ratiometric, synergistic combinations of hydrophilic, and hydrophobic anticancer drugs. These LbL NPs circumvent some of the challenges of conventional drug delivery approaches and provide greater tumor remediation, prolonged blood half‐life, targeted delivery, and colocalization of multiple anticancer agents within tumor tissue while avoiding nonspecific release of cytotoxic compounds in healthy tissues at pH 7.4. In addition to overcoming drug‐resistance mechanisms, LbL NPs reduce systemic toxicity. Although not investigated here, LbL NPs are also an ideal platform for the codelivery of cytotoxic agents in combination with nucleic acids, such as siRNA or microRNA, that can modulate specific genetic pathways in tumors to elicit strong cytotoxic and immunomodulatory responses.^32^, ^70^, ^71^ The translational potential of these systems in clinical settings will be enhanced by the use of known liposomal formulations and nonimmunogenic biomacromolecules, such as polysaccharides and polypeptide backbones, as well as recent advances in the scale‐up and potential manufacture of these systems.^41^, ^72^, ^73^ Targeted genetic testing and an expanded understanding of pathophysiological variations among individual patients will further guide the design of this modular nanomedicine platform toward personalized cancer therapy, particularly for the critical challenge of ovarian cancer.

## 4 METHODS

### 4.1 Cell culture

All ovarian cancer cell lines were gifts from Dr. Ronny Drapkin (Perelman School of Medicine, University of Pennsylvania, Philadelphia, PA), and luciferized^74^ except A431, which was obtained from ATCC (Manassas, VA). All cell lines were cultured in RPMI1640 (Cellgro, Manassas, VA), except COV362 and COV318, which were cultured in DMEM, JHOS2, and Kuramochi, which were cultured in DMEM/Ham's F‐12 50/50. BMN 673 and AZD2281 were purchased from MedChem Express (Princeton, NJ), dissolved in DMSO, and stored in aliquots at −20°C. Cisplatin was purchased from Sigma‐Aldrich (St. Louis, MO).

### 4.2 LbL polymeric liposomal NP assembly

Liposomes were formulated at a mass ratio of 56:39:5 using 1,2‐distearoyl‐*sn*‐glycero‐3‐phosphocholine (DSPC), 1‐palmitoyl‐2‐oleoyl‐*sn*‐glycero‐3‐phospho‐(1′‐rac‐glycerol) sodium salt (POPG; both from Avanti Polar Lipids), in a final concentration of 25 mg/mL and cholesterol (Sigma‐Aldrich), thus 14.24 mg of DSCP; 9.5 mg of cholesterol and 1.25 mg of POPG dissolved in chloroform and methanol in a 3.33:1.67 mL volume ratio together with the hydrophobic drug BMN 673 or AZD2281 at 12 wt% of each. The ratio of lipid to drug was 3 mg for BMN 673 and 3 mg for AZD2281. A thin film was formed under rotary evaporation at 40°C and 145 mbar, desiccated overnight, and then hydrated with 5 mL 300 mM of citric acid buffer (pH 4.0) for 1 hr in a water bath at 65°C under sonication of 5 mL of liposomes preperation. The warm liposomes were passed through 0.45‐ and 0.2‐μm polyethersulfone (PES)‐syringe filters, and the pH of the liposomes was adjusted to 6.5 by 300 mM sodium carbonate buffer. The final liposome concentration after pH adjustment was 20 mg/mL. Cisplatin was loaded into the liposomes core at 10 mg/mL in 0.9% sodium chloride solution. The final preparation was filtered via a 0.2‐μm PES syringe filter. The PARPi accumulated within the liposomes, from an equal mass supply (3 mg of each drug per 50 mg of lipid used) during fabrication, and the encapsulation efficiency was higher for AZD2281 than for BMN 673.

Salt buffers and excess unloaded drug were removed using a TFF (mPES MidiKros filter module #DO2‐E750‐05‐N) system,^41^ followed by LbL assembly of liposomal NPs with poly‐l‐lysine (PLL) and HA. PLL HBr (15–30 kDa, Sigma‐Aldrich) and HA (200 kDa, Lifecore) were used as received from the vendor. All solutions were sterile filtered with a 0.2‐μM filter prior to use. The liposomes suspension was diluted to 1 mg/mL and added dropwise to 45 mL of 500 μM PLL with rapid stirring, followed by TFF purification. The concentrated PLL‐layered liposomes were diluted to 1 mg/mL and added dropwise to a rapidly stirring 45‐mL solution of HA (10 μM), followed by stirring for 30 min at 4°C. The HA‐layered liposomes were recovered by TFF wash as described previously.^41^

### 4.3 Physicochemical characterization

The hydrodynamic size and PDI were determined using DLS (Malvern ZS90 particle analyzer, *λ* = 633 nm, material/dispersant RI 1.590/1.330). The zeta potential was determined using laser Doppler electrophoresis (Malvern ZS90). All samples were diluted in MilliQ water. Quantification of BMN 673 and AZD2281 in the NPs was determined by high performance liquid chromatography‐mass spectrometry (HPLC‐MS) (1,100 series triple quadruple LC/MS, Agilent Technologies, Lexington, MA) equipped with UV–VIS detector. An aliquot of 10 μL of each sample was injected in a mobile phase of 1:1 acetonitrile/water (pH 5.0) 0.1% formic acid and separated with C16, 3 μL 2.1 × 150, 120A separation column between absorbance of 180–240 nm. BMN 673 and AZD2281 were detected at 210 and 220 nm, respectively. The cisplatin concentration was determined by ICP‐MS 7900 (Agilent Technologies, Lexington, MA). Samples were initially prepared in 1:1 acetonitrile/water solution to release cisplatin from NPs and further diluted (1:200) MilliQ water and 5 μL of each sample analyzed on ICP‐MS and compared to known cisplatin standard. Cryogenic TEM was performed on a JEOL 2100 FEG instrument to image a frozen dilute sample of the liposomal suspension at 120 kV.

### 4.4 Drug release study, extraction, and quantification

Dual drug‐loaded LbL NPs were incubated under sink conditions (1‐L sink for 1 mL of liposome suspension) in 1× PBS pH 7.4 or in citrate buffer pH 5.0 under agitation in 1 mL of 3500 MWCO Float‐A‐Lyzer (Spectrum) at 37°C. PBS or citrated buffer was replenished with the equivalent of sample taken each day of the experiment. Samples were taken of the liposomes to quantify remaining drug concentrations by for quantification. For quantification of cumulative release of AZD2281 and BMN 673 samples were vortex in 50:50 mixture of mobile phase of acetonitrile:water with 0.1% formic acid (pH 5). Samples were first separated by HPLC and analyzed by mass spectrometry. The separation was between absorbance of 180 and 240 nm for BMN 673 and 210 and 220 nm for AZD2281. For cumulative release cisplatin samples quantification, samples were diluted 200‐fold in MilliQ water and platinum content measured on ICP‐MS (Agilent Technologies). All samples were analyzed in triplicate.

### 4.5 Cell viability and immunofluorescence

Cells were cultured at 5,000 cells/well in a 96‐well plate in triplicate for 24 hr, followed by treatment with varying doses of free or encapsulated drugs alone or in combination. Cell viability was determined at 96 hr using the CellTitre Glo assay (Promega) on a Tecan microplate reader. Immunofluorescence was performed as previously described.^75^ Briefly, to detect HR, COV362, OVCAR4, and OVCAR8 cells were plated at 6.0 × 10^4^ cells on 18‐mm PLL‐coated glass cover sides in 12‐well plates for 24 hr and then treated with 10 μM free or encapsulated drugs for 24 hr. The cells were costained with anti‐rabbit RAD51 (H‐92, sc‐8349, Santa Cruz Biotechnology, TX) and anti‐mouse phosphorylated γH2AX (Ser139), (#JBW301, Millipore, CA) primary antibodies overnight at 4°C. Protein expression was detected with anti‐rabbit IgG Alexa Fluor 647 and anti‐mouse IgG Alexa Fluor 488 (A‐21121, Invitrogen) secondary antibodies with DAPI (0.1 mg/mL) nuclear counterstaining for 2 hr at ambient temperature. Images were acquired on an Applied Precision Delta Vision Microscope (Olympus).

### 4.6 Western blotting

Western blotting was performed using a standard protocol.^76^ The following primary antibodies were used at a dilution of 1:1,000: BRCA1 (D54A8, #9025), BRCA2 rabbit (H‐300, sc‐8326, #9012), CD44 mouse (8E2, #5640), PTEN (D4.3, #9188), EGFR rabbit, ß‐actin mouse, and ß‐actin rabbit (13E5, #4979; all Cell Signaling Technology, Danvers, MA). Anti‐rabbit IgG horseradish peroxidase (HRP)‐conjugated secondary antibody (#7074; Cell Signaling Technology) was used. Protein expression was visualized with ECL prime western blotting detection reagent substrate on an Image Quant LAS4000 imager.

### 4.7 Pharmacokinetics and targeting of ovarian tumor cells

Four‐ to six‐week‐old female immunocompetent BALB/c mice (Taconic) were IV and IP injected with 100 μL of blank Cy5.5‐PLL‐conjugated and HA terminal‐layered liposomal NPs (2.5 mg/mL). Fluorescence signals were obtained for the whole body and for harvested organs on a Xenogen IVIS imaging system (Caliper) at various time points (*n* = 3). For blood half‐life determination, retro‐orbital bleeds were performed on a set of animals (*n* = 3). All signals were normalized to the Cy5.5 autofluorescence signal, and the fluorescence signal from the NP content in the blood circulation was used to calculate and fit a two‐compartmental pharmacokinetics model in the PRISM GraphPad software v5. To target ovarian tumors in vivo, COV362‐mCherry cells (3 × 10^6^ cells) were IP implanted in female NCR nude mice. Three weeks after tumor induction, xenograft tumor‐ and nontumor‐bearing mice were injected with Cy5.5‐PLL‐conjugated and HA terminal‐layered NPs. d‐luciferin (100uL of 15mg/mL) was IP administered, and IVIS was used to simultaneously image the Cy5.5‐NP biodistribution and luciferase signal at various time points (excitation at 675 nm for Cy5.5 and 720 nm for luciferase).

### 4.8 Maximum tolerated dose

To determine the therapeutic dose for in vivo treatment, we performed high and medium maximum tolerated dose studies with free or encapsulated cisplatin and BMN 673 or AZD2281 alone or in combination. Animals were doses with free and nano‐formulated drugs were administrated weekly by IV injections of the following agents: PBS or blank liposomal NP control (5.0 mg/kg once weekly), free cisplatin (7.00 mg/kg in 0.9% NaCl solution, free AZD2281 (50 mg/kg), free BMN 673 (0.33 mg/kg), free cisplatin and AZD2281 (5.00/50 mg/kg), free cisplatin and BMN 673 (5.0/0.33 mg/kg), and nano‐formulated drugs at: cisplatin (5.00 mg/kg), free AZD2281 (50 mg/kg), free BMN 673 (0.33 mg/kg), free cisplatin and AZD2281 (5.00/50 mg/kg), and free cisplatin and BMN 673 (5.00/0.33 mg/kg). Four‐ to six‐week‐old female NCR nude mice (nu/nu, Taconic) were weighed and randomized to 12 treatment arms (*n* = 3 per group) in both the free and encapsulated dosing cohorts. The therapeutic agents were IV administered via the tail vein. Weight loss and lethargy or morbidity were assessed daily for 15 days. Body weight was measured daily, and an approximately 60‐μL retro‐orbital blood sample was taken weekly in a 0.2‐mL K3E K3EDTA minicollect tube (VWR) for complete blood count (cbc) on a Vetscan HM5 hematology analyzer (Abaxis) to assess treatment efficacy and bone marrow toxicity.

### 4.9 Mouse xenograft studies

The number of mice required in each treatment group to achieve statistical significance was determined by a power calculation.^77^ Female NCR (nu/nu, Taconic, NY) mice were intraperitoneally injected with luciferase‐expressing OVCAR8 cells (2 × 10^6^ cells/200 μL) suspended in sterile Hank balanced salt solution. After 2–3 weeks, tumor‐bearing mice were randomized into 12 groups (*n* = 8–10 mice for the free and encapsulated drug treatment groups, *n* = 15 for the PBS and liposomes alone control). Free and encapsulated drugs were administered weekly by IV injection of the following agents: PBS, drug carriers, 10% 2‐hydroxyl‐propyl‐beta‐cyclodextrine, 6% solutol, and 84% PBS for free olaparib and BMN 673^28^ or blank liposomal NP control (5.0 mg/kg) once weekly); free cisplatin (6 mg/kg); free AZD2281 (50 mg/kg); free BMN 673 (0.35 mg/kg); free cisplatin and AZD2281 (5/50 mg/kg); free cisplatin and BMN 673 (5/0.35 mg/kg); encapsulated cisplatin (5 mg/kg); encapsulated AZD2281 (50 mg/kg); encapsulated BMN 673 (0.33 mg/kg); encapsulated cisplatin and AZD2281 (5/46.8 mg/kg); and encapsulated cisplatin and BMN 673 (5/0.32 mg/kg). Once weekly, the mice were weighed, and bioluminescence signals of tumor growth kinetics were obtained by IP injection of 0.1 mL of d‐luciferin (30 mg/kg for 10 min) followed by animal imaging on a Xenogen IVIS Imaging system (Caliper). All animal experimentations adhere to the National Institute of Health (NIH) guide for the care and use of laboratory animals and procedures were also conducted with the approval of the MIT Committee on Animal Care (CAC).

### 4.10 Necropsy and immunohistochemistry

Animals were monitored daily and euthanized when they became moribund, when their body weight decreased by more than 15%, or when lethargy, ruffled fur, or severe ascites were observed. Upon euthanasia, an arterial blood sample was withdrawn, and the body weight and volume of ascites were recorded. Complete liver and kidney biochemistry tests were performed using serum by Charles River Laboratories (Shrewsbury, MA) to assess organ toxicity. Tissue specimens were immediately fixed in formalin for paraffin embedding or snap frozen in optimal cutting medium (Miles, Inc., Elkhart, IN) for frozen slide preparation. All immunohistochemistry and H&E staining and sample processing were performed by the Swanson Technology histology core (MIT, Cambridge, MA). All samples were cut into 5‐mm‐thick sections and stained with H&E for gross organ histological analysis. For immunohistochemistry, tissue sections were stained with the following primary anti‐human antibodies: cleaved Caspase 3 rabbit (Asp175 #96640), anti‐Ki67 mouse (8D5, #9449), anti‐cleaved PARP rabbit (#56625), anti‐phospho‐histone H2A.X rabbit (Ser139, #9718), and PAX8 mouse monoclonal antibody. The secondary antibody was a mouse‐on‐mouse HRP‐polymer antibody (MM620L, BIOCARE Medical, Pacheco, CA). All staining was quantified by two investigators in a blinded fashion. The histology slides were independently reviewed and scored by Dr. Roderick Bronson (faculty at Tufts Veterinary School, Jackson Laboratory and Harvard Medical School) and a member of the MIT Swanson histology core.

### 4.11 Statistical analysis

Cell analysis was based upon triplicate experiments, and the results are presented as the mean ± *SEM* of at least three independent experiments. Student's *t* test was used for comparisons between two groups, and one‐way ANOVA was used for comparisons among three or more groups of in vitro and in vivo data. Differences between samples were considered statistically significant at *p <* .05. Two‐way ANOVA was used to compare histoscore for foci after treatments. Survival data were analyzed using the Kaplan–Meier method, and log‐rank statistics was used to analyze the survival distribution. All statistical analyses were performed using the GraphPad Prism6 software (GraphPad Prism 5.0, GraphPad Software, La Jolla, CA).

## CONFLICTS OF INTEREST

The authors declare no potential conflicts of interest.

## AUTHOR CONTRIBUTIONS

L.B.M., S.W.M., M.A.Q., K.M.E., J.L., P.P.G., and P.T.H. were involved in conception and design. L.B.M., S.W.M., J.L., H.X., E.P., M.A.Q., K.M.E., P.P.G., J.L., and P.T.H. contributed to the development of methodology. L.B.M., S.W.M., J.L., H.X., M.A.Q., K.M.E., E.P., A.K.R., P.P.G., J.L., and P.T.H. were involved in the analysis and interpretation of data (e.g., statistical analysis, biostatistics, computational analysis). L.B.M., S.W.M., J.L., B.N., H.X., M.A.Q., K.M.E., J.L., P.P.G., and P.T.H. wrote, reviewed, and/or revised the manuscript. L.B.M., J.L., and P.T.H. were involved in administrative, technical, or material support (i.e., reporting or organizing data, constructing databases). P.T.H. supervised the study.

## Supporting information


**Figure S1** AZD2281, BMN 673 and cisplatin dose–response curves. Dose–response curves of **A**, Kuramochi **B**, OVISE and **C**, OVCAR4 ovarian cancer cell lines treated with free AZD2281, BMN 673 or cisplatin. **D**, Top panel, detection and bottom panel, quantification of RAD51, γH2AX foci formation and DAPI by immunostaining in COV362 cells after treatment for 24 h with 1 μM AZD2281, cisplatin or BMN 673. The data are presented as the mean ± SEM of at least three independent experiments. Statistical significance was determined by one‐way ANOVA with Bonferroni's multiple comparison tests; * *p* < 0.05, ** *p* < 0.01, *** *p* < 0.001.
**Figure S2.** The escalating drug dose studies was better tolerated when delivered in encapsulated form versus the free drugs. **A,** Body weight; **B**, hemoglobin (Hb); **C**, platelets; and **D**, total white blood cells (WBC) were measured 2 weeks after IV injection of NCR nude female mice on three consecutive days with single monotherapy of AZD2281, BMN 673 or cisplatin or with cisplatin combined with AZD2881 or cisplatin‐BMN 673 as the free drugs (FD, left panel) or nanoparticles (NP, right panel). The data were normalized to untreated mice, analyzed as the area under the curve (AUC), and plotted as histograms. The data are presented as the mean ± SEM, n = 3. Statistical analysis was performed by one‐way ANOVA; ** *p* < 0.01, *** *p* < 0.001. FD denotes free drug and NP, encapsulated nanoparticles.
**Figure S3**. HA terminal‐layered polymeric liposomal nanoparticles produce a superior in vivo therapeutic response in HGSOC xenografts. NCR nude female mice bearing luciferase‐ and CD44‐expressing OVCAR8 xenografts were treated weekly via IV administration of vehicle, AZD2281, BMN 673 or cisplatin monotherapies or cisplatin combined with AZD2281 or BMN 673 (n = 8). **A,B** Plots of the bioluminescent signal flux of the tumors from the start of treatment. Statistical significance was determined by one‐way ANOVA with Turkey's multiple comparison tests. **C,D**, Kaplan–Meier plots of survival fractions. Statistical significance was determined using the log‐rank (Mantel‐Cox) test. **E,F**, The body weight distribution of the treatment groups was measured and graphed as scatter plots. Statistical significance was determined by one‐way ANOVA with Bonferroni's multiple comparison tests. Data are presented as the mean ± SEM; * *p* < 0.05, ** *p* < 0.01, ***p < 0.001. **G**, Schematic illustration of the design of the polymeric liposomal nanoparticle assembly with loaded therapeutic cargo and the treatment mechanism. FD, denote free and NP encapsulated nanoparticles.
**Figure S4.** Control tissues for H&E staining. Light micrograph of tumors and tissues from control mice showing histological immunohistochemistry staining controls for cleaved PARP (cPARP) (left panel), γH2AX(pSer139) foci formation (middle panel) and cleaved caspase 3 (CC3) expression (right panel). Scale bar, 200 μm.

## References

[1] Rabinerson D , Kaplan B , Levavi H , Neri A . The biology of ovarian cancer of epithelial origin. Isr J Med Sci. 1996;32(11):1128‐1133. 8960089

[2] Tortolero‐Luna G , Mitchell MF . The epidemiology of ovarian cancer. J Cell Biochem Suppl. 1995;23:200‐207. 8747397 10.1002/jcb.240590927

[3] Pisano C , Bruni GS , Facchini G , Marchetti C , Pignata S . Treatment of recurrent epithelial ovarian cancer. Ther Clin Risk Manag. 2009;5(4):421‐426. 19753136 10.2147/tcrm.s4317 PMC2695243

[4] Kurman RJ . Origin and molecular pathogenesis of ovarian high‐grade serous carcinoma. Ann. Oncol. 2013;24(Suppl 10):x16‐x21. 24265397 10.1093/annonc/mdt463

[5] Safra T . Hereditary ovarian cancer: biology, response to chemotherapy and prognosis. Womens Health. 2009;5(5):543‐553. 10.2217/whe.09.40 19702453

[6] Romanini A , Tanganelli L , Carnino F , et al. First‐line chemotherapy with epidoxorubicin, paclitaxel, and carboplatin for the treatment of advanced epithelial ovarian cancer patients. Gynecol Oncol. 2003;89(3):354‐359. 12798695 10.1016/s0090-8258(03)00128-8

[7] Ozols RF . Systemic therapy for ovarian cancer: current status and new treatments. Semin Oncol. 2006;33(2 Suppl 6):S3‐S11. 10.1053/j.seminoncol.2006.03.011 16716797

[8] Sessa C , Del Conte G . Targeted therapies: tailored treatment for ovarian cancer: are we there yet? Nat Rev Clin Oncol. 2010;7(2):80‐82. 20118979 10.1038/nrclinonc.2009.233

[9] Mantia‐Smaldone GM , Edwards RP , Vlad AM . Targeted treatment of recurrent platinum‐resistant ovarian cancer: current and emerging therapies. Cancer Management and Research. 2011;3:25‐38. 21734812 10.2147/CMR.S8759 PMC3130354

[10] McLachlan J , Banerjee S . Olaparib for the treatment of epithelial ovarian cancer. Expert Opin Pharmacother. 2016;17:995‐1003. 26967466 10.1517/14656566.2016.1165205

[11] Talazoparib bests chemo for breast cancer. Cancer Discov. 2018;8(2):OF3. 10.1158/2159-8290.CD-NB2017-180 29242215

[12] Weil MK , Chen AP . PARP inhibitor treatment in ovarian and breast cancer. Curr Probl Cancer. 2011;35(1):7‐50. 21300207 10.1016/j.currproblcancer.2010.12.002 PMC3063418

[13] Drew Y . The development of PARP inhibitors in ovarian cancer: from bench to bedside. Br J Cancer. 2015;113(Suppl 1):S3‐S9. 26669452 10.1038/bjc.2015.394 PMC4816267

[14] Thorsell AG , Ekblad T , Karlberg T , et al. Structural basis for potency and promiscuity in poly(ADP‐ribose) polymerase (PARP) and tankyrase inhibitors. J Med Chem. 2017;60(4):1262‐1271. 28001384 10.1021/acs.jmedchem.6b00990 PMC5934274

[15] Kim G , Ison G , McKee AE , et al. FDA approval summary: olaparib monotherapy in patients with deleterious germline BRCA‐mutated advanced ovarian cancer treated with three or more lines of chemotherapy. Clin Cancer Res. 2015;21(19):4257‐4261. 26187614 10.1158/1078-0432.CCR-15-0887

[16] Ison G , Howie LJ , Amiri‐Kordestani L , et al. FDA approval summary: niraparib for the maintenance treatment of patients with recurrent ovarian cancer in response to platinum‐based chemotherapy. Clin Cancer Res. 2018;24:4066‐4071. 29650751 10.1158/1078-0432.CCR-18-0042

[17] Balasubramaniam S , Beaver JA , Horton S , et al. FDA approval summary: rucaparib for the treatment of patients with deleterious BRCA mutation‐associated advanced ovarian cancer. Clin Cancer Res. 2017;23(23):7165‐7170. 28751443 10.1158/1078-0432.CCR-17-1337

[18] Rottenberg S , Jaspers JE , Kersbergen A , et al. High sensitivity of BRCA1‐deficient mammary tumors to the PARP inhibitor AZD2281 alone and in combination with platinum drugs. Proc Natl Acad Sci U S A. 2008;105(44):17079‐17084. 18971340 10.1073/pnas.0806092105 PMC2579381

[19] Hay T , Matthews JR , Pietzka L , et al. Poly(ADP‐ribose) polymerase‐1 inhibitor treatment regresses autochthonous Brca2/p53‐mutant mammary tumors in vivo and delays tumor relapse in combination with carboplatin. Cancer Res. 2009;69(9):3850‐3855. 19383921 10.1158/0008-5472.CAN-08-2388

[20] Pujade‐Lauraine E , Ledermann JA , Selle F , et al. Olaparib tablets as maintenance therapy in patients with platinum‐sensitive, relapsed ovarian cancer and a BRCA1/2 mutation (SOLO2/ENGOT‐Ov21): a double‐blind, randomised, placebo‐controlled, phase 3 trial. Lancet Oncol. 2017;18(9):1274‐1284. 28754483 10.1016/S1470-2045(17)30469-2

[21] Oza AM , Cibula D , Benzaquen AO , et al. Olaparib combined with chemotherapy for recurrent platinum‐sensitive ovarian cancer: a randomised phase 2 trial. Lancet Oncol. 2015;16(1):87‐97. 25481791 10.1016/S1470-2045(14)71135-0

[22] Ledermann JA , Harter P , Gourley C , et al. Overall survival in patients with platinum‐sensitive recurrent serous ovarian cancer receiving olaparib maintenance monotherapy: an updated analysis from a randomised, placebo‐controlled, double‐blind, phase 2 trial. Lancet Oncol. 2016;17(11):1579‐1589. 27617661 10.1016/S1470-2045(16)30376-X

[23] Wang B , Chu D , Feng Y , Shen Y , Aoyagi‐Scharber M , Post LE . Discovery and characterization of (8S,9R)‐5‐fluoro‐8‐(4‐fluorophenyl)‐9‐(1‐methyl‐1H‐1,2,4‐triazol‐5‐yl)‐2,7,8,9‐te trahydro‐3H‐pyrido[4,3,2‐de]phthalazin‐3‐one (BMN 673, Talazoparib), a novel, highly potent, and orally efficacious poly(ADP‐ribose) polymerase‐1/2 inhibitor, as an anticancer agent. J Med Chem. 2016;59(1):335‐357. 26652717 10.1021/acs.jmedchem.5b01498

[24] de Bono J , Ramanathan RK , Mina L , et al. Phase I, dose‐escalation, two‐part trial of the PARP inhibitor talazoparib in patients with advanced germline BRCA1/2 mutations and selected sporadic cancers. Cancer Discov. 2017;7(6):620‐629. 28242752 10.1158/2159-8290.CD-16-1250 PMC5905335

[25] Wilding G , Caruso R , Lawrence TS , et al. Retinal toxicity after high‐dose cisplatin therapy. J Clin Oncol. 1985;3(12):1683‐1689. 4067616 10.1200/JCO.1985.3.12.1683

[26] Rebmann U , Oehlmann U , Warnack W . Cisplatin‐induced nephrotoxic side effects of cytostatic chemotherapy of testicular tumors. Z Exp Chir Transplant Kunstliche Organe. 1990;23(2):83‐85. 1703720

[27] Balmana J , Tung NM , Isakoff SJ , et al. Phase I trial of olaparib in combination with cisplatin for the treatment of patients with advanced breast, ovarian and other solid tumors. Ann Oncol. 2014;25(8):1656‐1663. 24827126 10.1093/annonc/mdu187

[28] Shen Y , Rehman FL , Feng Y , et al. BMN 673, a novel and highly potent PARP1/2 inhibitor for the treatment of human cancers with DNA repair deficiency. Clin Cancer Res. 2013;19(18):5003‐5015. 23881923 10.1158/1078-0432.CCR-13-1391 PMC6485449

[29] Dhawan MS , Bartelink IH , Aggarwal RR , et al. Differential toxicity in patients with and without DNA repair mutations: phase I study of carboplatin and talazoparib in advanced solid tumors. Clin Cancer Res. 2017;23(21):6400‐6410. 28790114 10.1158/1078-0432.CCR-17-0703

[30] Lee JM , Hays JL , Chiou VL , et al. Phase I/Ib study of olaparib and carboplatin in women with triple negative breast cancer. Oncotarget. 2017;8(45):79175‐79187. 29108297 10.18632/oncotarget.16577 PMC5668030

[31] Mateo J , Moreno V , Gupta A , et al. An adaptive study to determine the optimal dose of the tablet formulation of the PARP inhibitor olaparib. Target Oncol. 2016;11(3):401‐415. 27169564 10.1007/s11523-016-0435-8

[32] Deng ZJ , Morton SW , Ben‐Akiva E , Dreaden EC , Shopsowitz KE , Hammond PT . Layer‐by‐layer nanoparticles for systemic codelivery of an anticancer drug and siRNA for potential triple‐negative breast cancer treatment. ACS Nano. 2013;7(11):9571‐9584. 24144228 10.1021/nn4047925 PMC3870477

[33] Morton SW , Lee MJ , Deng ZJ , et al. A nanoparticle‐based combination chemotherapy delivery system for enhanced tumor killing by dynamic rewiring of signaling pathways. Sci Signal. 2014;7(325):ra44. 24825919 10.1126/scisignal.2005261 PMC4138219

[34] Correa S , Dreaden EC , Gu L , Hammond PT . Engineering nanolayered particles for modular drug delivery. J Control Release. 2016;240:364‐386. 26809005 10.1016/j.jconrel.2016.01.040 PMC6450096

[35] Zhang RX , Wong HL , Xue HY , Eoh JY , Wu XY . Nanomedicine of synergistic drug combinations for cancer therapy—strategies and perspectives. J Control Release. 2016;240:489‐503. 27287891 10.1016/j.jconrel.2016.06.012 PMC5064882

[36] Dreaden EC , Kong YW , Morton SW , et al. Tumor‐targeted synergistic blockade of MAPK and PI3K from a layer‐by‐layer nanoparticle. Clin Cancer Res. 2015;21(19):4410‐4419. 26034127 10.1158/1078-0432.CCR-15-0013 PMC4624301

[37] Gu L , Deng ZJ , Roy S , Hammond PT . A combination RNAi‐chemotherapy layer‐by‐layer nanoparticle for systemic targeting of KRAS/P53 with cisplatin to treat non‐small cell lung cancer. Clin Cancer Res. 2017;23(23):7312‐7323. 28912139 10.1158/1078-0432.CCR-16-2186 PMC5712246

[38] Poon Z , Chang D , Zhao XY , Hammond PT . Layer‐by‐layer nanoparticles with a pH‐sheddable layer for in vivo targeting of tumor hypoxia. ACS Nano. 2011;5(6):4284‐4292. 21513353 10.1021/nn200876f PMC3125426

[39] Gelmon KA , Tischkowitz M , Mackay H , et al. Olaparib in patients with recurrent high‐grade serous or poorly differentiated ovarian carcinoma or triple‐negative breast cancer: a phase 2, multicentre, open‐label, non‐randomised study. Lancet Oncol. 2011;12(9):852‐861. 21862407 10.1016/S1470-2045(11)70214-5

[40] Yasukawa M , Fujihara H , Fujimori H , et al. Synergetic effects of PARP inhibitor AZD2281 and cisplatin in oral squamous cell carcinoma in vitro and in vivo. Int J Mol Sci. 2016;17(3):272. 26927065 10.3390/ijms17030272 PMC4813136

[41] Correa S , Choi KY , Dreaden EC , et al. Highly scalable, closed‐loop synthesis of drug‐loaded, layer‐by‐layer nanoparticles. Adv Funct Mater. 2016;26(7):991‐1003. 27134622 10.1002/adfm.201504385 PMC4847955

[42] Sosulski A , Horn H , Zhang L , et al. CD44 splice variant v8‐10 as a marker of serous ovarian cancer prognosis. PLoS One. 2016;11(6):e0156595. 10.1371/journal.pone.0156595 PMC4890777 27253518

[43] Ween MP , Oehler MK , Ricciardelli C . Role of versican, hyaluronan and CD44 in ovarian cancer metastasis. Int J Mol Sci. 2011;12(2):1009‐1029. 21541039 10.3390/ijms12021009 PMC3083686

[44] Justus CR , Dong L , Yang LV . Acidic tumor microenvironment and pH‐sensing G protein‐coupled receptors. Front Physiol. 2013;4:354. 24367336 10.3389/fphys.2013.00354 PMC3851830

[45] Helleday T . The underlying mechanism for the PARP and BRCA synthetic lethality: clearing up the misunderstandings. Mol Oncol. 2011;5(4):387‐393. 21821475 10.1016/j.molonc.2011.07.001 PMC5528309

[46] Dasari S , Tchounwou PB . Cisplatin in cancer therapy: molecular mechanisms of action. Eur J Pharmacol. 2014;740:364‐378. 25058905 10.1016/j.ejphar.2014.07.025 PMC4146684

[47] Stordal B , Timms K , Farrelly A , et al. BRCA1/2 mutation analysis in 41 ovarian cell lines reveals only one functionally deleterious BRCA1 mutation. Mol Oncol. 2013;7(3):567‐579. 23415752 10.1016/j.molonc.2012.12.007 PMC4106023

[48] Murai J , Huang SY , Renaud A , et al. Stereospecific PARP trapping by BMN 673 and comparison with olaparib and rucaparib. Mol Cancer Ther. 2014;13(2):433‐443. 24356813 10.1158/1535-7163.MCT-13-0803 PMC3946062

[49] Paull TT , Rogakou EP , Yamazaki V , Kirchgessner CU , Gellert M , Bonner WM . A critical role for histone H2AX in recruitment of repair factors to nuclear foci after DNA damage. Curr Biol. 2000;10(15):886‐895. 10959836 10.1016/s0960-9822(00)00610-2

[50] Wilkerson PM , Dedes KJ , Samartzis EP , et al. Preclinical evaluation of the PARP inhibitor BMN‐673 for the treatment of ovarian clear cell cancer. Oncotarget. 2017;8(4):6057‐6066. 28002809 10.18632/oncotarget.14011 PMC5351612

[51] Busschots S , O'Toole S , O'Leary JJ , Stordal B . Carboplatin and taxol resistance develops more rapidly in functional BRCA1 compared to dysfunctional BRCA1 ovarian cancer cells. Exp Cell Res. 2015;336(1):1‐14. 25499884 10.1016/j.yexcr.2014.12.001

[52] Tarsounas M , Davies D , West SC . BRCA2‐dependent and independent formation of RAD51 nuclear foci. Oncogene. 2003;22(8):1115‐1123. 12606939 10.1038/sj.onc.1206263

[53] Oliver FJ , de la Rubia G , Rolli V , Ruiz‐Ruiz MC , de Murcia G , Murcia JM . Importance of poly(ADP‐ribose) polymerase and its cleavage in apoptosis. Lesson from an uncleavable mutant. J Biol Chem. 1998;273(50):33533‐33539. 9837934 10.1074/jbc.273.50.33533

[54] Wang AZ , Langer R , Farokhzad OC . Nanoparticle delivery of cancer drugs. Annu Rev Med. 2012;63:185‐198. 21888516 10.1146/annurev-med-040210-162544

[55] Blanco E , Shen H , Ferrari M . Principles of nanoparticle design for overcoming biological barriers to drug delivery. Nat Biotechnol. 2015;33(9):941‐951. 26348965 10.1038/nbt.3330 PMC4978509

[56] Petras I , Magin RL . Simulation of drug uptake in a two compartmental fractional model for a biological system. Commun Nonlinear Sci Numer Simul. 2011;16(12):4588‐4595. 21822359 10.1016/j.cnsns.2011.02.012 PMC3150575

[57] Kim GH , Won JE , Byeon Y , et al. Selective delivery of PLXDC1 small interfering RNA to endothelial cells for anti‐angiogenesis tumor therapy using CD44‐targeted chitosan nanoparticles for epithelial ovarian cancer. Drug Deliv. 2018;25(1):1394‐1402. 29890852 10.1080/10717544.2018.1480672 PMC6096458

[58] Yang X , Iyer AK , Singh A , et al. MDR1 siRNA loaded hyaluronic acid‐based CD44 targeted nanoparticle systems circumvent paclitaxel resistance in ovarian cancer. Sci Rep. 2015;5:8509. 25687880 10.1038/srep08509 PMC4330541

[59] Journo‐Gershfeld G , Kapp D , Shamay Y , Kopecek J , David A . Hyaluronan oligomers‐HPMA copolymer conjugates for targeting paclitaxel to CD44‐overexpressing ovarian carcinoma. Pharm Res. 2012;29(4):1121‐1133. 22350800 10.1007/s11095-012-0672-1

[60] Mitra AK , Davis DA , Tomar S , et al. In vivo tumor growth of high‐grade serous ovarian cancer cell lines. Gynecol Oncol. 2015;138(2):372‐377. 26050922 10.1016/j.ygyno.2015.05.040 PMC4528621

[61] Liu JF , Tolaney SM , Birrer M , et al. A phase 1 trial of the poly(ADP‐ribose) polymerase inhibitor olaparib (AZD2281) in combination with the anti‐angiogenic cediranib (AZD2171) in recurrent epithelial ovarian or triple‐negative breast cancer. Eur J Cancer. 2013;49(14):2972‐2978. 23810467 10.1016/j.ejca.2013.05.020 PMC3956307

[62] Fuse E , Kobayashi T , Inaba M , Sugiyama Y . Prediction of the maximal tolerated dose (MTD) and therapeutic effect of anticancer drugs in humans: integration of pharmacokinetics with pharmacodynamics and toxicodynamics. Cancer Treat Rev. 1995;21(2):133‐157. 7758004 10.1016/0305-7372(95)90024-1

[63] McCluskey LL , Dubeau L . Biology of ovarian cancer. Curr Opin Oncol. 1997;9(5):465‐470. 9327225 10.1097/00001622-199709050-00011

[64] Perets R , Wyant GA , Muto KW , et al. Transformation of the fallopian tube secretory epithelium leads to high‐grade serous ovarian cancer in Brca;Tp53;Pten models. Cancer Cell. 2013;24(6):751‐765. 24332043 10.1016/j.ccr.2013.10.013 PMC3917315

[65] Laury AR , Hornick JL , Perets R , et al. PAX8 reliably distinguishes ovarian serous tumors from malignant mesothelioma. Am J Surg Pathol. 2010;34(5):627‐635. 20414098 10.1097/PAS.0b013e3181da7687

[66] Gao Z , Li Z , Yan J , Wang P . Irinotecan and 5‐fluorouracil‐co‐loaded, hyaluronic acid‐modified layer‐by‐layer nanoparticles for targeted gastric carcinoma therapy. Drug des Devel Ther. 2017;11:2595‐2604. 10.2147/DDDT.S140797 PMC5592948 28919710

[67] Liu X , Han F , Zhao P , Lin C , Wen X , Ye X . Layer‐by‐layer self‐assembled multilayers on PEEK implants improve osseointegration in an osteoporosis rabbit model. Nanomedicine. 2017;13(4):1423‐1433. 28131883 10.1016/j.nano.2017.01.011

[68] Rodriguez‐Rodriguez L , Sancho‐Torres I , Mesonero C , Gibbon DG , Shih WJ , Zotalis G . The CD44 receptor is a molecular predictor of survival in ovarian cancer. Med Oncol. 2003;20(3):255‐263. 14514975 10.1385/MO:20:3:255

[69] Fong PC , Boss DS , Yap TA , et al. Inhibition of poly(ADP‐ribose) polymerase in tumors from BRCA mutation carriers. N Engl J Med. 2009;361(2):123‐134. 19553641 10.1056/NEJMoa0900212

[70] Tangutoori S , Spring BQ , Mai Z , Palanisami A , Mensah L , Hasan T . Simultaneous delivery of cytotoxic and biologic therapeutics using nanophotoactivatable liposomes enhances treatment efficacy in a mouse model of pancreatic cancer. Nanomedicine. 2015;12:223‐234. 26390832 10.1016/j.nano.2015.08.007 PMC4728029

[71] Dreaden EC , Morton SW , Shopsowitz KE , Deng ZJ , Yaffe MB , Hammond PT . Self‐assembled polymer drug carriers for rational combination and RNA interference therapy of solid tumors. Abstr Pap Am Chem S. 2014(1);248.

[72] Morton SW , Herlihy KP , Shopsowitz KE , et al. Scalable manufacture of built‐to‐order nanomedicine: spray‐assisted layer‐by‐layer functionalization of PRINT nanoparticles. Adv Mater. 2013;25(34):4707‐4713. 23813892 10.1002/adma.201302025 PMC4040353

[73] Becker AL , Johnston AP , Caruso F . Layer‐by‐layer‐assembled capsules and films for therapeutic delivery. Small. 2010;6(17):1836‐1852. 20715072 10.1002/smll.201000379

[74] Elias KM , Emori MM , Papp E , et al. Beyond genomics: critical evaluation of cell line utility for ovarian cancer research. Gynecol Oncol. 2015;139(1):97‐103. 26321251 10.1016/j.ygyno.2015.08.017 PMC4587360

[75] Quadir MA , Morton SW , Mensah LB , et al. Ligand‐decorated click polypeptide derived nanoparticles for targeted drug delivery applications. Nanomedicine. 2017;13:1797‐1808. 28263813 10.1016/j.nano.2017.02.010 PMC5641973

[76] Rizvi I , Gurkan UA , Tasoglu S , et al. Flow induces epithelial‐mesenchymal transition, cellular heterogeneity and biomarker modulation in 3D ovarian cancer nodules. Proc Natl Acad Sci U S A. 2013;110(22):E1974‐E1983. 23645635 10.1073/pnas.1216989110 PMC3670329

[77] Lenth RV . Statistical power calculations. J Anim Sci. 2007;85(13 Suppl):E24‐E29. 17060421 10.2527/jas.2006-449

