# Supplementary material for: Layer‐by‐layer nanoparticles for novel delivery of cisplatin and PARP inhibitors for platinum‐based drug resistance therapy in ovarian cancer
Source: Bioeng Transl Med. 2019 Jun 14;4(2):e10131. doi: 10.1002/btm2.10131 (PMC6584097; doi:10.1002/btm2.10131)

**Supplementary Figure Legends**

**Figure S1.** AZD2281, BMN 673 and cisplatin dose-response curves. Dose-response curves of **A**, Kuramochi **B**, OVISE and **C**, OVCAR4 ovarian cancer cell lines treated with free AZD2281, BMN 673 or cisplatin. **D**, Top panel, detection and bottom panel, quantification of RAD51, γH2AX foci formation and DAPI by immunostaining in COV362 cells after treatment for 24 h with 1 μM AZD2281, cisplatin or BMN 673. The data are presented as the mean ± SEM of at least three independent experiments. Statistical significance was determined by one-way ANOVA with Bonferroni’s multiple comparison tests; * *P*<0.05, ** *P*<0.01, *** *P*<0.001.

**Figure S2.** The escalating drug dose studies was better tolerated when delivered in encapsulated form versus the free drugs. **A,** Body weight; **B**, hemoglobin (Hb); **C**, platelets; and **D**, total white blood cells (WBC) were measured 2 weeks after IV injection of NCR nude female mice on three consecutive days with single monotherapy of AZD2281, BMN 673 or cisplatin or with cisplatin combined with AZD2881 or cisplatin BMN 673 as the free drugs (FD, left panel) or nanoparticles (NP, right panel). The data were normalized to untreated mice, analyzed as the area under the curve (AUC), and plotted as histograms. The data are presented as the mean ± SEM, n=3. Statistical analysis was performed by one-way ANOVA; ** *P*<0.01, *** *P*<0.001. FD denotes free drug and NP, encapsulated nanoparticles.

**Figure S3**. HA terminal-layered polymeric liposomal nanoparticles produce a superior *in vivo* therapeutic response in HGSOC xenografts*.* NCR nude female mice bearing luciferase- and CD44-expressing OVCAR8 xenografts were treated weekly via IV administration of vehicle, AZD2281, BMN 673 or cisplatin monotherapies or cisplatin combined with AZD2281 or BMN 673 (n=8). **A,B** Plots of the bioluminescent signal flux of the tumors from the start of treatment. Statistical significance was determined by one-way ANOVA with Turkey’s multiple comparison tests. **C,D**, Kaplan-Meier plots of survival fractions. Statistical significance was determined using the log-rank (Mantel-Cox) test. **E,F**, The body weight distribution of the treatment groups was measured and graphed as scatter plots. Statistical significance was determined by one-way ANOVA with Bonferroni’s multiple comparison tests. Data are presented as the mean ± SEM; * *P*<0.05, ** *P*<0.01, *** *P*<0.001. **G**, Schematic illustration of the design of the polymeric liposomal nanoparticle assembly with loaded therapeutic cargo and the treatment mechanism. FD, denote free and NP encapsulated nanoparticles.

**Figure S4.** Control tissues for H&E staining. Light micrograph of tumors and tissues from control mice showing histological immunohistochemistry staining controls for cleaved PARP (cPARP) (left panel), γH2AX(pSer139) foci formation (middle panel) and cleaved caspase 3(CC3) expression (right panel). Scale bar, 200 μm.

SUPPLEMENTARY FIGURES

FIGURE S1


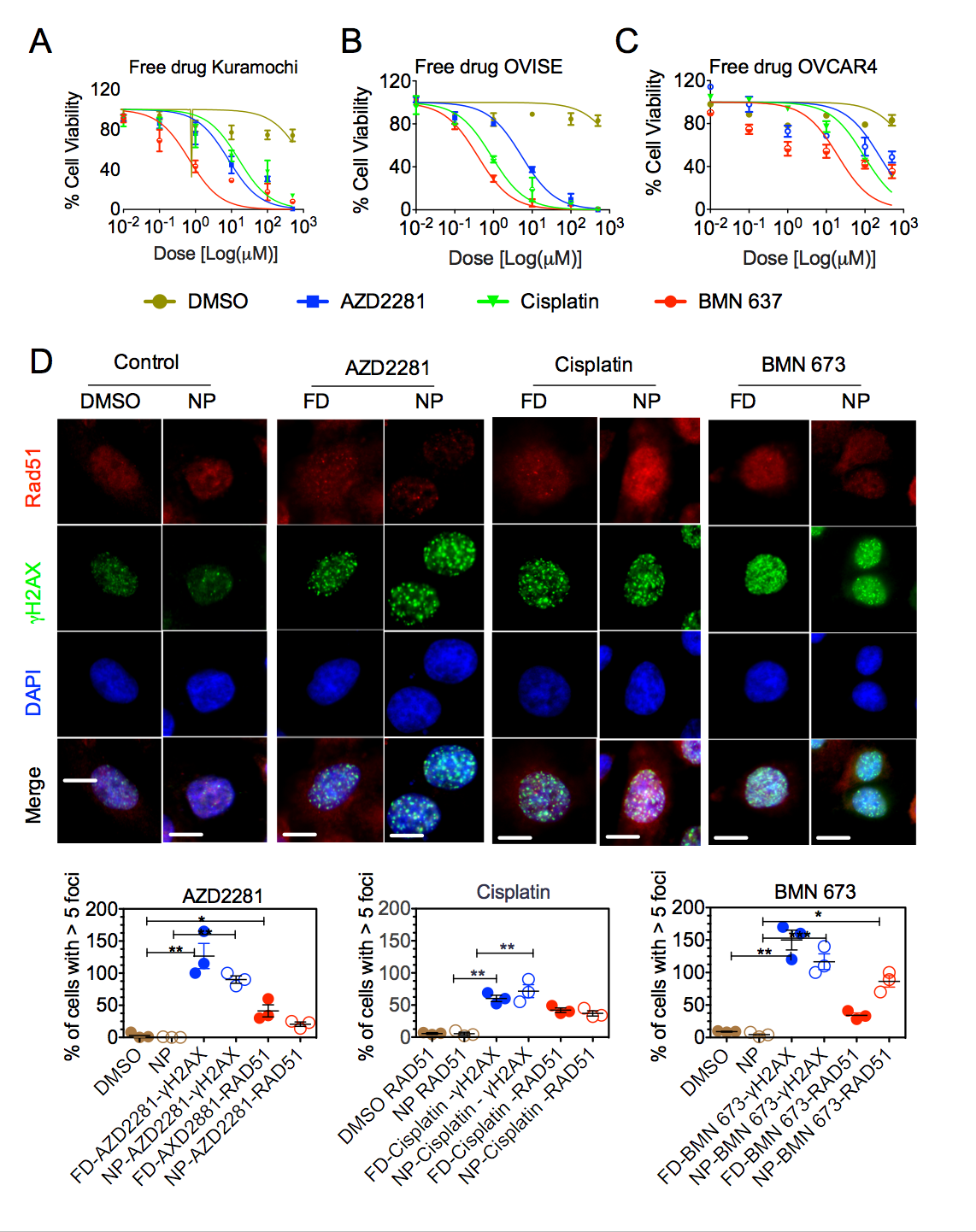


FIGURE S2


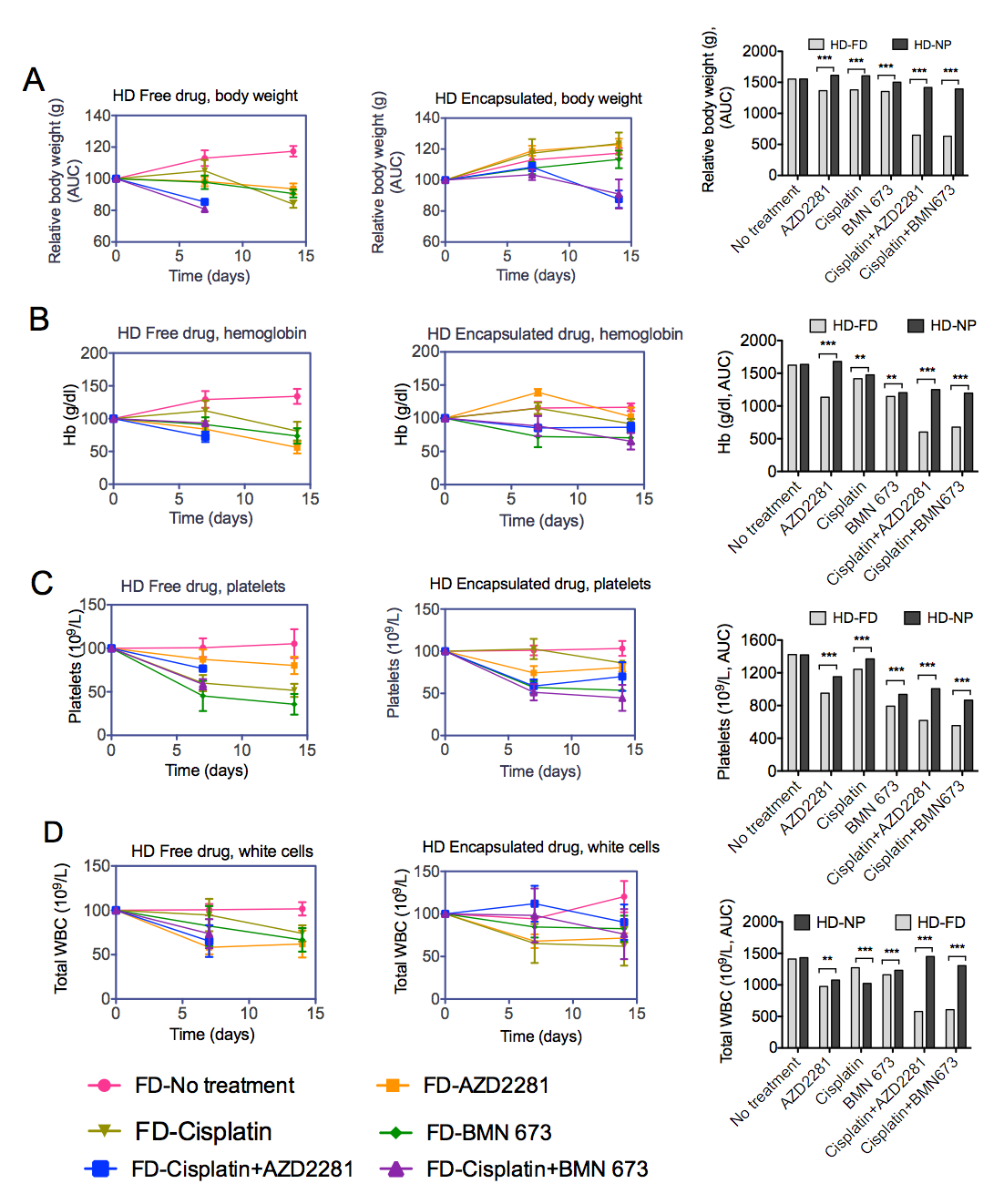


FIGURE S3


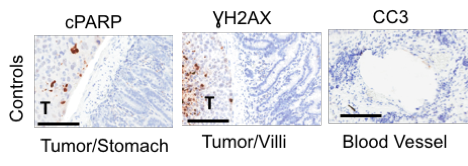


FIGURE S4


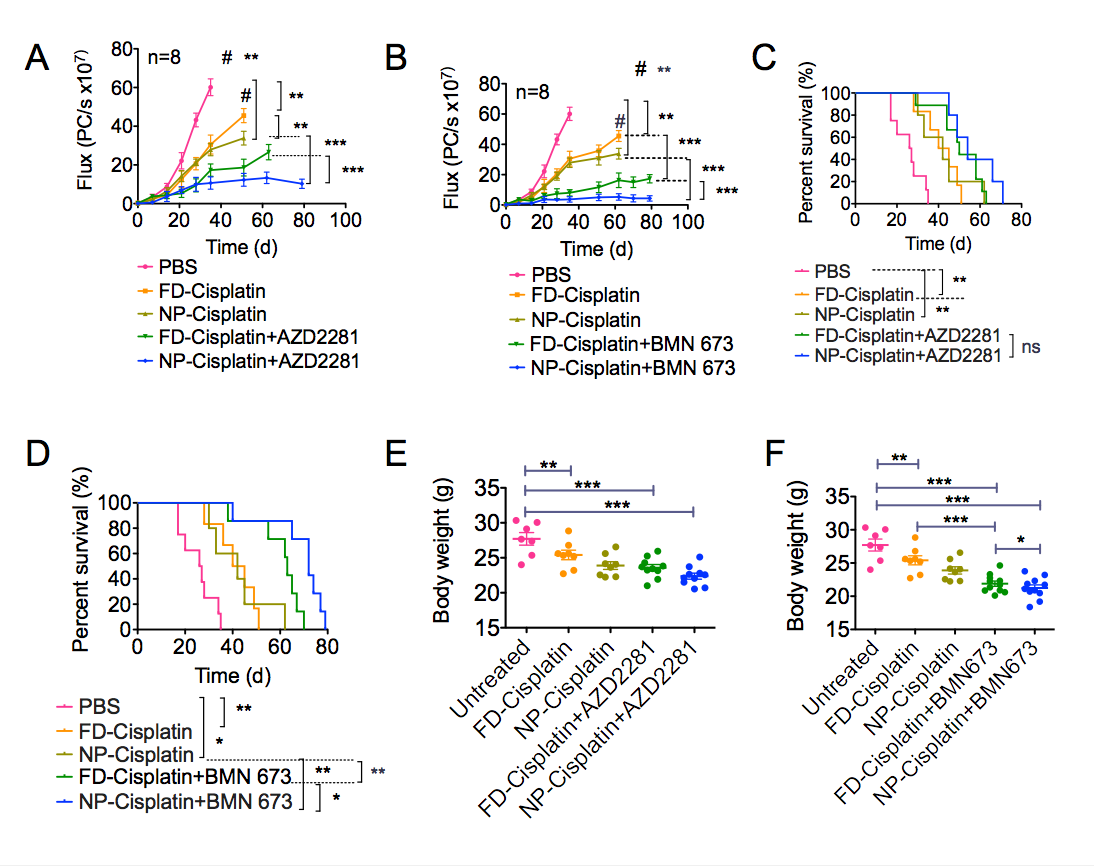

Supplement: Supplementary file 1 — Figure S1 AZD2281, BMN 673 and cisplatin dose–response curves. Dose–response curves of A, Kuramochi B, OVISE and C, OVCAR4 ovarian cancer cell lines treated with free AZD2281, BMN 673 or cisplatin. D, Top panel, detection and bottom panel, quantification of RAD51, γH2AX foci formation and DAPI by immunostaining in COV362 cells after treatment for 24 h with 1 μM AZD2281, cisplatin or BMN 673. The data are presented as the mean ± SEM of at least three independent experiments. Statistical significance was determined by one‐way ANOVA with Bonferroni's multiple comparison tests; * p < 0.05, ** p < 0.01, *** p < 0.001. Figure S2. The escalating drug dose studies was better tolerated when delivered in encapsulated form versus the free drugs. A, Body weight; B, hemoglobin (Hb); C, platelets; and D, total white blood cells (WBC) were measured 2 weeks after IV injection of NCR nude female mice on three consecutive days with single monotherapy of AZD2281, BMN 673 or cisplatin or with cisplatin combined with AZD2881 or cisplatin‐BMN 673 as the free drugs (FD, left panel) or nanoparticles (NP, right panel). The data were normalized to untreated mice, analyzed as the area under the curve (AUC), and plotted as histograms. The data are presented as the mean ± SEM, n = 3. Statistical analysis was performed by one‐way ANOVA; ** p < 0.01, *** p < 0.001. FD denotes free drug and NP, encapsulated nanoparticles. Figure S3. HA terminal‐layered polymeric liposomal nanoparticles produce a superior in vivo therapeutic response in HGSOC xenografts. NCR nude female mice bearing luciferase‐ and CD44‐expressing OVCAR8 xenografts were treated weekly via IV administration of vehicle, AZD2281, BMN 673 or cisplatin monotherapies or cisplatin combined with AZD2281 or BMN 673 (n = 8). A,B Plots of the bioluminescent signal flux of the tumors from the start of treatment. Statistical significance was determined by one‐way ANOVA with Turkey's multiple comparison tests. C,D, Kaplan–Meier plots of survival [file BTM2-4-na-s001.docx]
